# Morphological, anatomical, and transcriptomics analysis reveals the regulatory mechanisms of cassava plant height development

**DOI:** 10.1186/s12864-024-10599-2

**Published:** 2024-07-17

**Authors:** Zhaoqin Cai, Lixia Ruan, Wanling Wei, Wen He, Haixia Yang, Huixian Chen, Zhenhua Liang, Zhenling Huang, Xiu Lan, Xiufen Zhang, Ruolan Huang, Chunhui Zhao, Tianyuan Li, Longfei He, Hengrui Li

**Affiliations:** 1 Guangxi South Subtropical Agricultural Science Research Institute, Nanning, 530007 PR China; 2 National Demonstration Center for Experimental Plant Science Education, College of Agriculture, Guangxi University, Nanning, 530004 PR China

**Keywords:** Plant height, Cassava, Transcriptome, Physiology, Anatomical structure

## Abstract

**Background:**

Cassava is one of three major potato crops and the sixth most important food crop globally. Improving yield remains a primary aim in cassava breeding. Notably, plant height significantly impacts the yield and quality of crops; however, the mechanisms underlying cassava plant height development are yet to be elucidated.

**Results:**

In this study, we investigated the mechanisms responsible for cassava plant height development using phenotypic, anatomical, and transcriptomic analyses. Phenotypic and anatomical analysis revealed that compared to the high-stem cassava cultivar, the dwarf-stem cassava cultivar exhibited a significant reduction in plant height and a notable increase in internode tissue xylem area. Meanwhile, physiological analysis demonstrated that the lignin content of dwarf cassava was significantly higher than that of high cassava. Notably, transcriptome analysis of internode tissues identified several differentially expressed genes involved in cell wall synthesis and expansion, plant hormone signal transduction, phenylpropanoid biosynthesis, and flavonoid biosynthesis between the two cassava cultivars.

**Conclusions:**

Our findings suggest that internode tissue cell division, secondary wall lignification, and hormone-related gene expression play important roles in cassava plant height development. Ultimately, this study provides new insights into the mechanisms of plant height morphogenesis in cassava and identifies candidate regulatory genes associated with plant height that can serve as valuable genetic resources for future crop dwarfing breeding.

**Supplementary Information:**

The online version contains supplementary material available at 10.1186/s12864-024-10599-2.

## Introduction

Plant height is a crucial trait influencing crop yield and quality. Notably, dwarf crop breeding is a key strategy used to enhance productivity. A reduction in plant height facilitates mechanized harvesting, increases yield and photosynthetic efficiency, enhances fertilizer tolerance, supports dense planting, improves lodging resistance, and enhances nutrient transport to reproductive organs. Moreover, dwarfing reduces the need for chemical growth regulators, thereby protecting the ecological environment. Therefore, understanding the regulatory mechanisms and genetic basis of plant height traits remains a major topic in genetic breeding research. This focus has been particularly extensive in rice, with the application of rice and wheat dwarf varieties significantly increasing crop yield in the 1960s and 1970s, leading to the Green Revolution [1].

Alongside environment factors, gene regulation plays a leading role in crop plant height development. For example, overexpression of the brassinosteroid (BR) degradation–related gene *AtBAT1* in transgenic creeping bentgrass was found to induce dwarfing, shorter internodes, smaller leaf angles, and other BR deficiency–related phenotypes [2]. Meanwhile, overexpression of *CYP94C2b* in rice increases internode length and plant height [3]. Similarly, the *GmLHY* gene regulates soybean plant height development through regulation of the gibberellin (GA) pathway [4]. In wheat, overexpression of *Rht1* and *Rht2* genes not only reduces plant height, but also increases tiller numbers and grains per spike, thereby significantly improving yield [1, 5].

Numerous studies have highlighted associations between crop dwarfing and deletions or mutations of genes involved in GA, BR, auxin (IAA), and strigolactone (SL) biosynthesis or signal transduction pathways [6]. For example, mutations in the *Arabidopsis* BRI1 (Brassinosteroid-Insensitive 1) gene, which encodes a receptor-like kinase, results in the inability to detect BR signals, ultimately leading to a severe dwarf phenotype [7]. In rice, mutations in the *sd1* gene, which encodes GA20ox, a key enzyme in the GA synthesis pathway, lead to a reduction in GA content and the shortening of internode length, resulting in a dwarf phenotype [8–10]. Similarly, deletion of rice IAA co-receptor genes *OsTIR1* and *OsAFB2-5* reduce plant height to varying degrees, accompanied by changes in yield, tillering, root traits [11]. Meanwhile, abnormal expression of the key IAA delivery genes ABCB1/PGP1 in the maize mutant brachytic2 (br2) and sorghum mutant dwarf3 (d3) resulted in a dwarfing phenotype [12]. SL has also been found to mediate rice plant height by regulating D53 degradation via the D14-D3 complex, thereby reversing the repression of downstream target genes [13].

Other factors influencing plant dwarfing include transcription factors and cell wall–associated genes. For example, CesA and CSL genes are responsible for cell wall biosynthesis, while expansin (EXP), and XTH genes influence cell wall loosening, thereby affecting cell growth during stem elongation [14]. Notably, plants with CesA or CSL mutations exhibit dwarf phenotypes [15–19]. Meanwhile inhibition of EXP gene expression results in reduced plant height, early flowering, and leaf curl [20–23]. Transcription factors can respond to both external environmental factors and endogenous hormone signals, regulating plant growth by influencing downstream target genes [24–26]. For example, overexpression of NAC transcription factors has been found to reduce abscisic acid (ABA) and BR content in apples, resulting in shorter internodes [27]. However, despite significant advances in understanding the genetic regulation of plant height and identifying several associated genes, the functions and regulatory mechanisms of many plant height–associated genes remains unclear due to the complex nature of this trait. Therefore, further research is needed to explore the roles and regulatory mechanisms of various genes influencing plant height.

Cassava (*Manihot esculenta* Crantz), a plant belonging to the *Manihot* genus in the Euphorbiaceae family, ranks among the world’s three top tuber crops alongside potato and sweet potato. With an annual yield exceeding 200 million tons, cassava serves as a staple food for over 700 million people worldwide, making it the sixth most important food crop globally. Beyond its role in food security, cassava serves as an important source of industrial raw material, feed, biomass energy, and food processing [28, 29]. Consequently, cassava has a strong reputation, being termed the “king of starch” and “energy crop”. Notably, cassava exhibits resilience to barren, dry, and acidic soil and can be planted on slope land. Moreover, this crop offers various advantages, including the primary use of asexual reproduction, high photosynthetic efficiency, extensive management, low cost, and high yield potential, making it a promising crop for industrial development. However, research on cassava plant height development remains sparse. Therefore, this study employed two cassava varieties with significant differences in height to investigate the mechanisms underlying cassava plant height development by analyzing morphological structure, anatomical structure, lignin content, cellulose content, and gene expression.

## Results

### Phenotypic characteristics, cytology, and physiology of XX048 and NZ199 plants

**Fig. 1:**
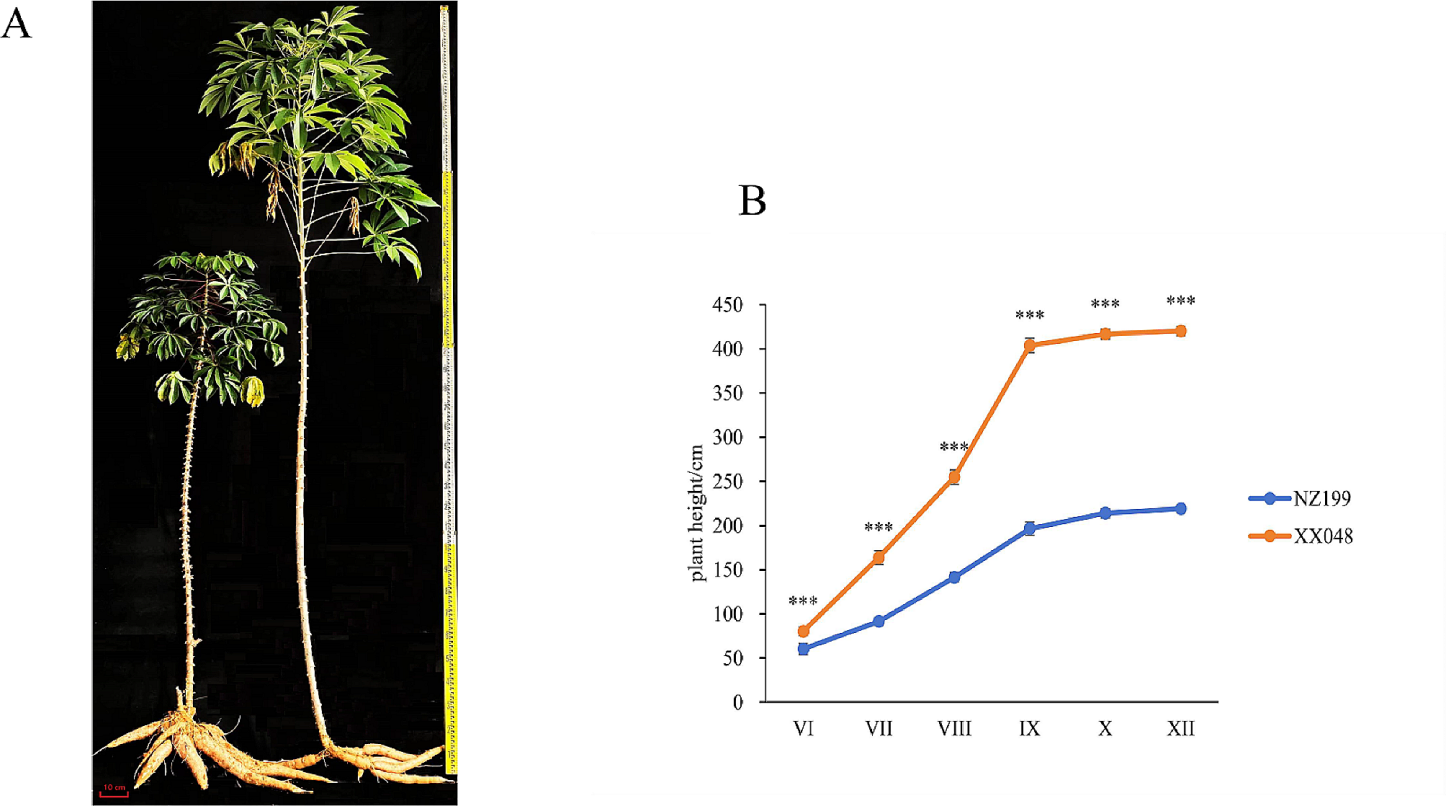
Comparison of the phenotypic characteristics of XX048 and NZ199 cassava plants. **A**: Plant heights of XX048 and NZ199 variants. Scale bars = 0.1 m. **B**: Plant heights of XX048 and NZ199 across six developmental stages; orange denotes NZ199, while blue represents XX048. Error bars indicate the SD. *** denotes a significant difference of *p* < 0.001

The plant heights of the two cassava varieties XX048 and NZ199 were 420.27 ± 5.2 cm and 219 ± 4.57 cm, respectively; this difference was determined to be statistically significant (Fig. 1). August–September is considered the period of rapid growth in cassava plant height. During this period, XX048 plant height increased by nearly 150 cm, while NZ199 plant height increased by approximately 55 cm. This indicated that XX048 plants grew significantly faster than NZ199 plants, with the difference in height between the two variants being the largest during this period.

**Fig. 2:**
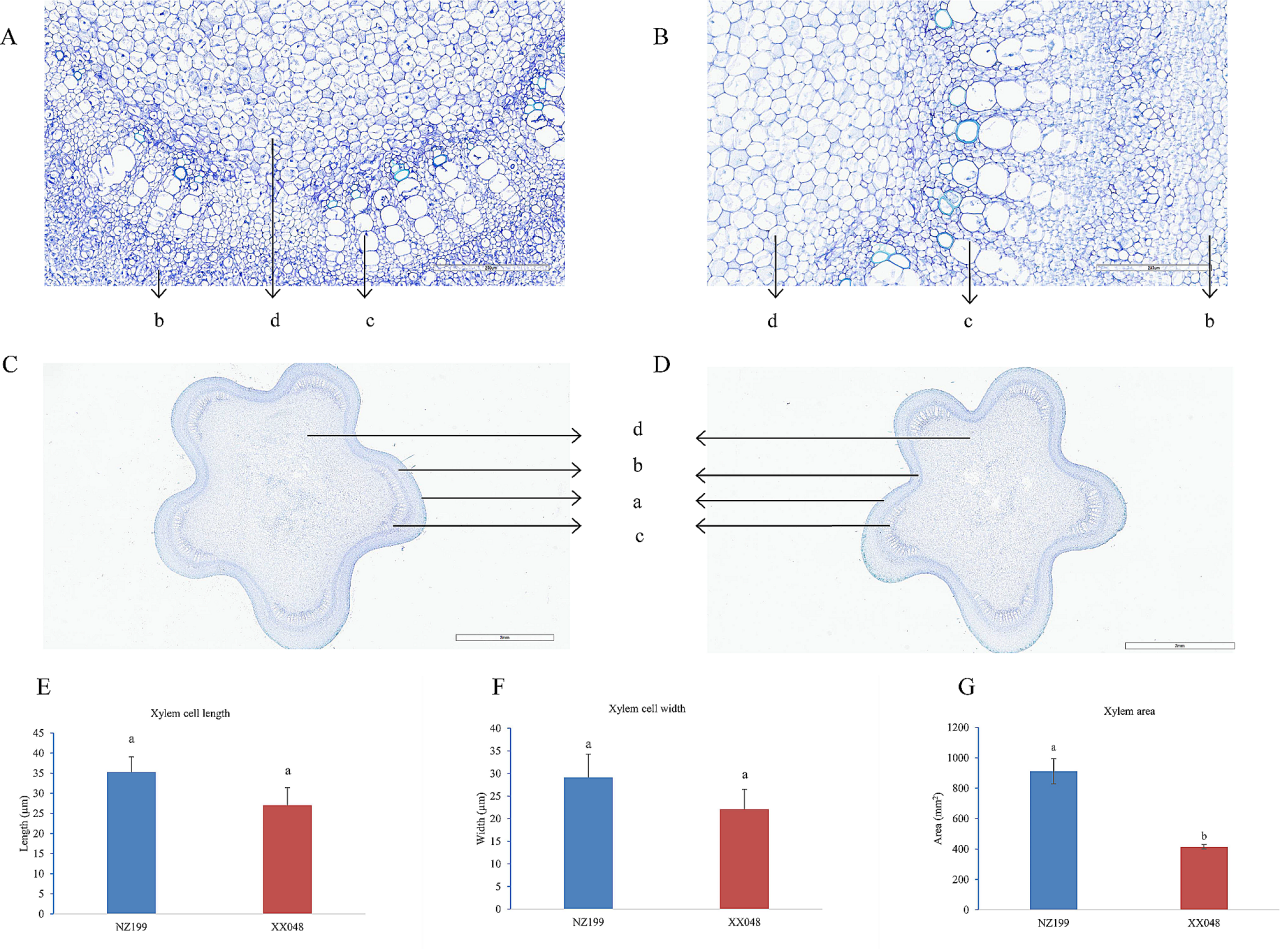
Anatomical characteristics of stem sections obtained from XX048 and NZ199 cassava plants. **A**-**D**: Transverse sections showing the **a**: epidermis, **b**: cortex, **c**: xylem, and **d**: microtubule column. **E**-**G** Analysis of stem sections in each variant. Error bars indicate the SD. Different letters denote significant differences between XX048 and NZ199 plants (*P* < 0.05)

To identify the anatomical differences between XX048 and NZ199 plants, we performed paraffin section staining of the topmost internode tissue. The woody area of XX048 was determined to be 414.87 ± 14.28 mm^2^, while that of NZ199 was 911.10 ± 82.62 mm^2^; notably, this difference in woody area was statistically significant. Meanwhile, the average length and width of XX048 xylem cells were 27.04 ± 4.37 mm and 22.11 ± 4.36 mm, respectively, while the average length and width of NZ199 xylem cells were 35.25 ± 3.81 mm and 29.12 ± 5.15 mm, respectively (Fig. 2). However, there was no significant difference observed between the length and width of xylem cells in XX048 and NZ199 plants. Overall, these results indicate that the xylem of NZ199 is more developed and has a higher degree of lignification.

**Fig. 3:**
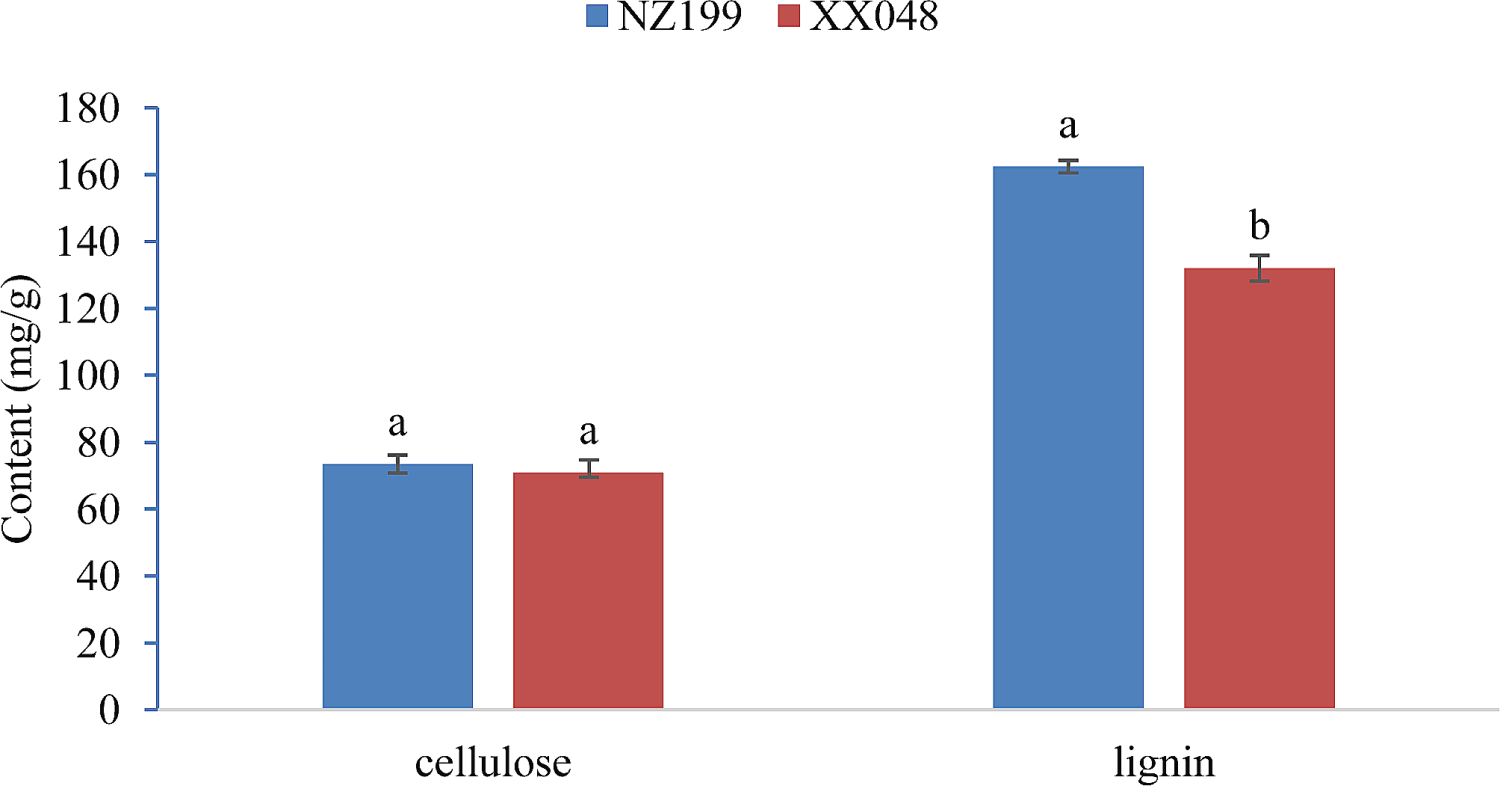
Differences in lignin and cellulose content between XX048 and NZ199 cassava plants. Error bars indicate the SD. Different letters associated with the same trait denote significant differences between XX048 and NZ199 variants (*P* < 0.05)

Next, we measured lignin and cellulose content in the topmost internode tissue of XX048 and NZ199 cassava plants (Fig. 3). The lignin content of NZ199 and XX048 plants is 162.37 ± 1.81 mg/g and 132.01 ± 3.83 mg/g, respectively. Meanwhile, the cellulose content of NZ199 and XX048 is 73.47 ± 2.69 mg/g and 70.91 ± 1.45 mg/g, respectively. Notably, the difference in lignin content between the two variants was determined to be statistically significant, while the difference in cellulose content was not significant. These results indicate differences in the cell wall lignification development in the two cassava variants, with the main stem of NZ199 cassava entering secondary wall lignification development earlier. Notably, these findings are consistent with the cytological results.

### Transcriptomic analysis

**Fig. 4:**
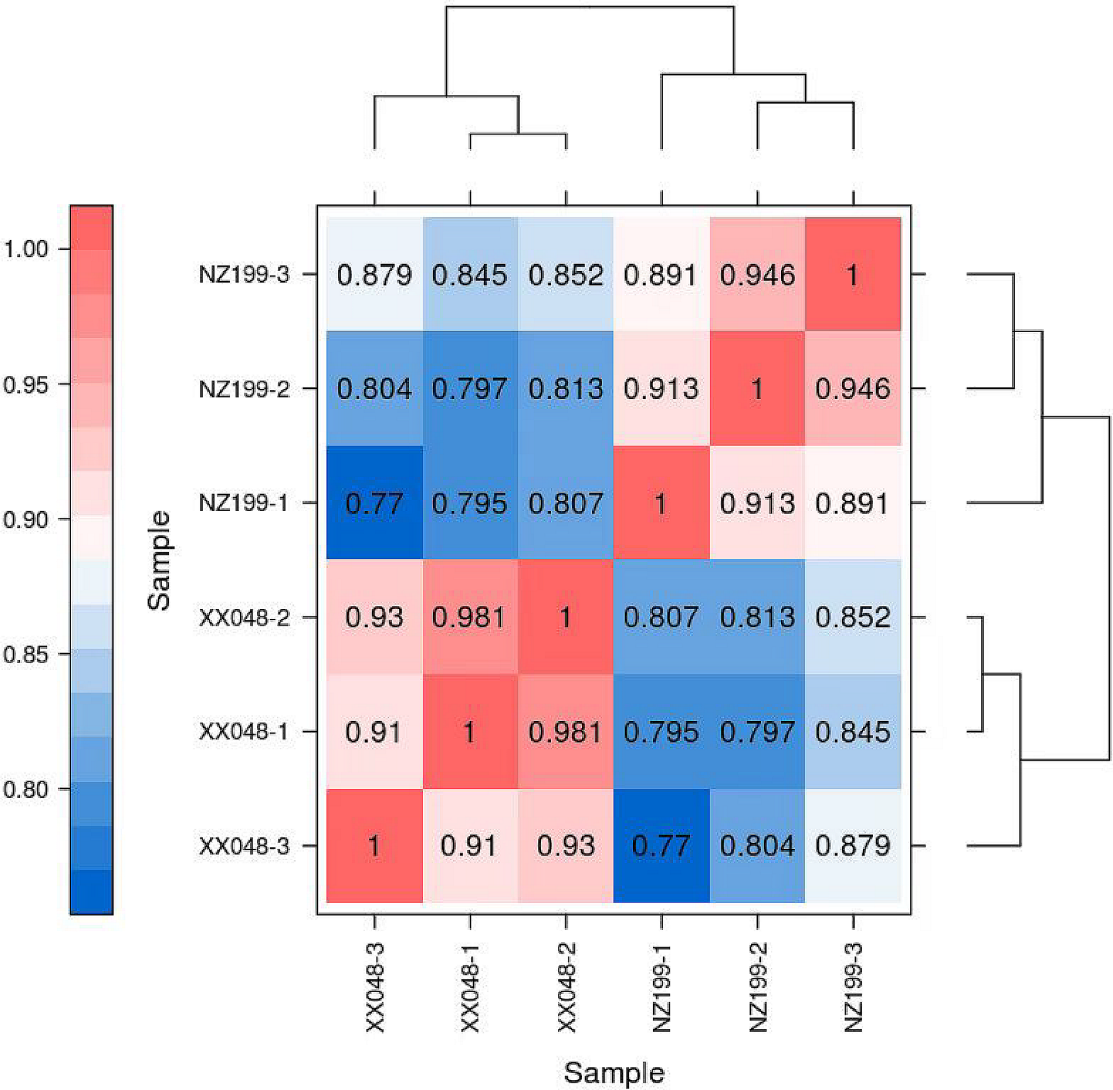
Correlation analysis between sample replicates of XX048 and NZ199 cassava variants

To explore the key genes linked to plant height development in cassava, six cDNA libraries were constructed. After removing splices and low-quality reads from Illumina sequencing data, 121,791,451 clean reads were obtained (Supplementary Table 1); the percentages of Q20 and Q30 base rates were > 97.82% and > 93.65%, respectively. The clean reads of each sample were then compared with the cassava reference genome, and the corresponding comparison efficiency ranged from 94.56 to 94.88%. The sample correlation heat map indicated that the R^2^ value among three biological repeat samples was > 0.89, with that of most of the samples being > 0.9, indicating that this experiment was highly reproducible and the data were reliable (Fig. 4).

**Fig. 5:**
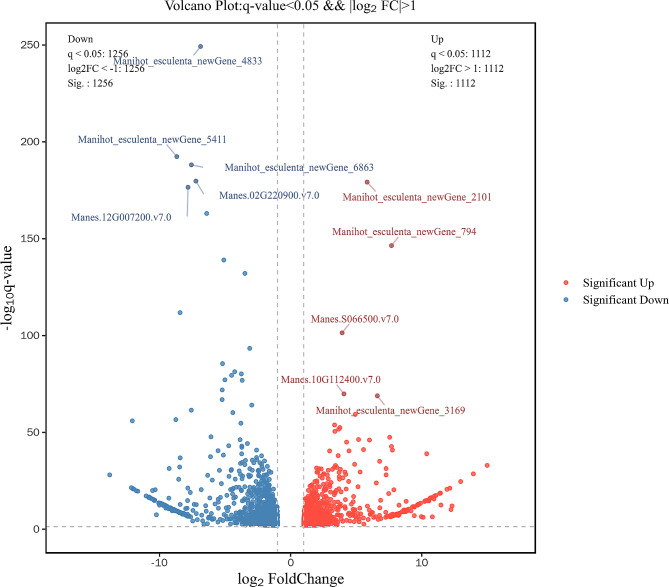
Volcano map showing differentially expressed gene expression between XX048 and NZ199 cassava plants

To investigate the expression pattern of genes associated with cassava plant height development, we compared the transcriptome maps of cassava with tall and short stems. Differentially expressed genes (DEGs) between cassava with tall and short stems were identified according to a log2 fold change ≥ 2 and a p – value < 0.01. A total of 2368 DEGs were identified between XX048 and NZ199 variants, of which 1112 genes were up-regulated and 1256 genes were down-regulated (Fig. 5).

**Fig. 6:**
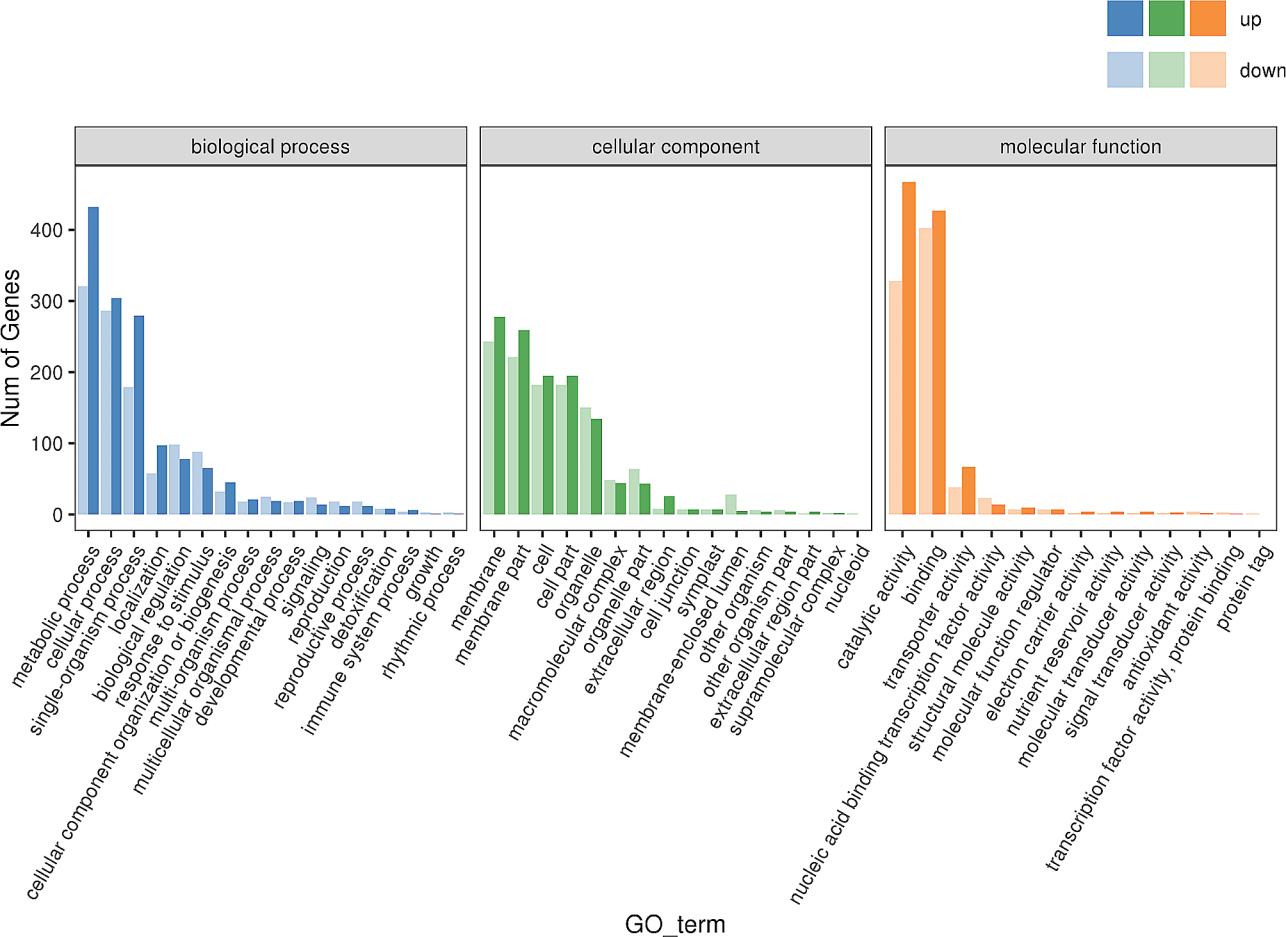
Gene Ontology (GO) classification of DEGs between XX048 and NZ199 cassava variants

To understand the biological processes associated with the identified DEGs, we classified DEGs into GO functions. These DEGs were categorized into three main GO functional groups (Fig. 6): biological process (BP), cellular component (CC), and molecular function (MF). The three most abundant sub-categories in the BP group were “metabolic process” (753 DEGs), “cellular process” (590 DEGs), and “single-organism process” (458 DEGs). In the CC group, the four most abundant sub-categories were “membrane” (521 DEGs), “membrane part” (480 DEGs), “cell part” (377 DEGs), and “cell” (377 DEGs). Meanwhile, the majority of DEGs in the MF group were associated with “binding” (829 DEGs), “catalytic activity” (795 DEGs), and “transporter activity” (105 DEGs).

**Fig. 7:**
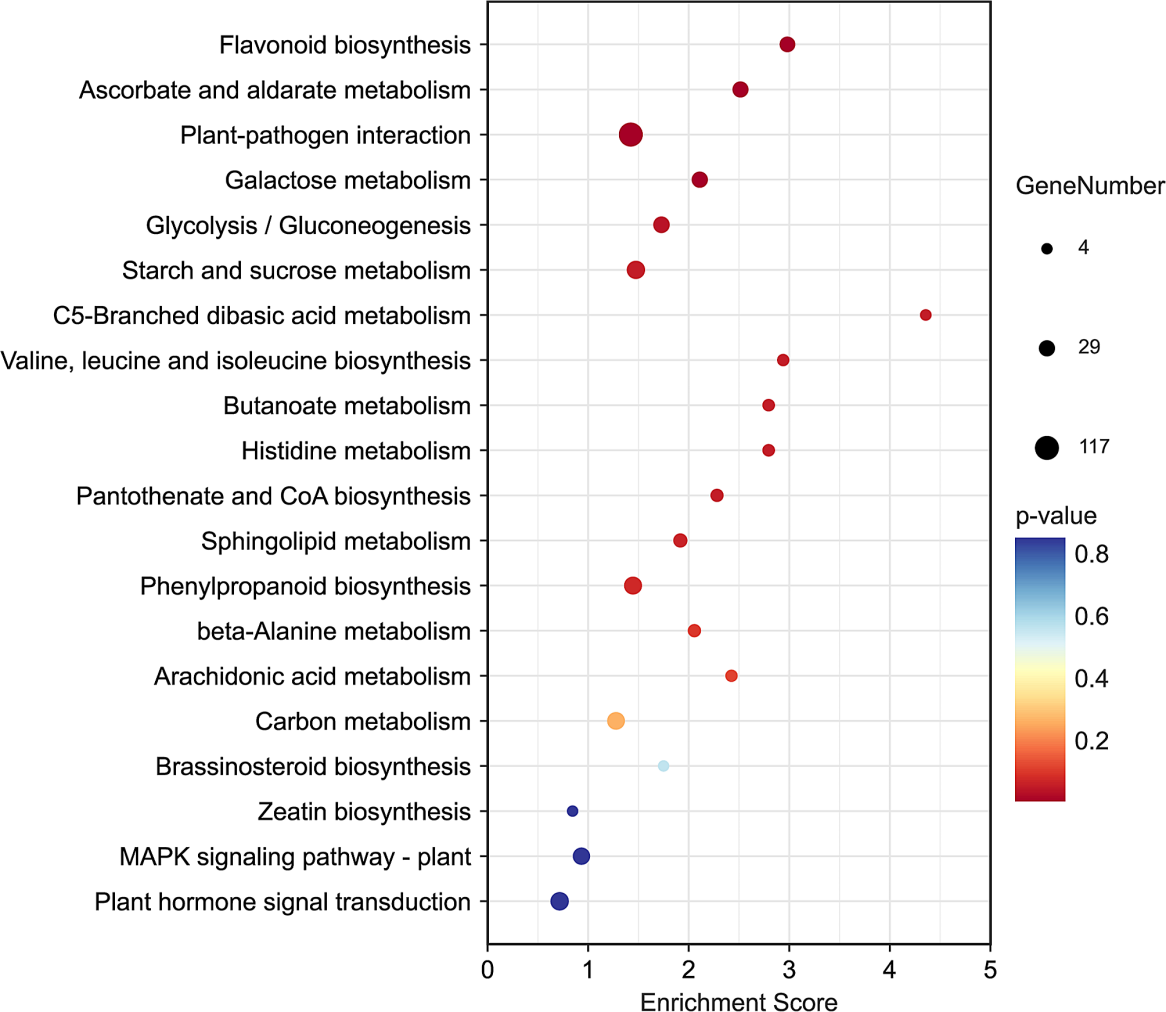
KEGG enrichment analysis of DEGs between XX048 and NZ199 cassava plant variants. The x axis denotes the enrichment score, which represents the ratio of DEGs identified in a specific pathway to all the genes contained in this pathway. The y axis denotes each KEGG pathway. Bubble size indicates the number of genes annotated to that specific KEGG Pathway

Finally, KEGG enrichment analysis revealed that the five most abundant pathways associated with these DEGs include “plant-pathogen interaction” (117 DEGs), “plant hormone signal transduction” (47 DEGs), “starch and sucrose metabolism” (46 DEGs), “phenylpropanoid biosynthesis” (43 DEGs), and “carbon metabolism” (41 DEGs). Notably, the “flavonoid biosynthesis”, “brassinosteroid biosynthesis”, and “zeatin biosynthesis” pathways were also enriched, with the flavonoid biosynthesis pathway being the most significantly enriched among these biosynthetic pathways (Fig. 7).

### DEGs associated with plant hormone biosynthesis and signal transduction pathways

**Fig. 8:**
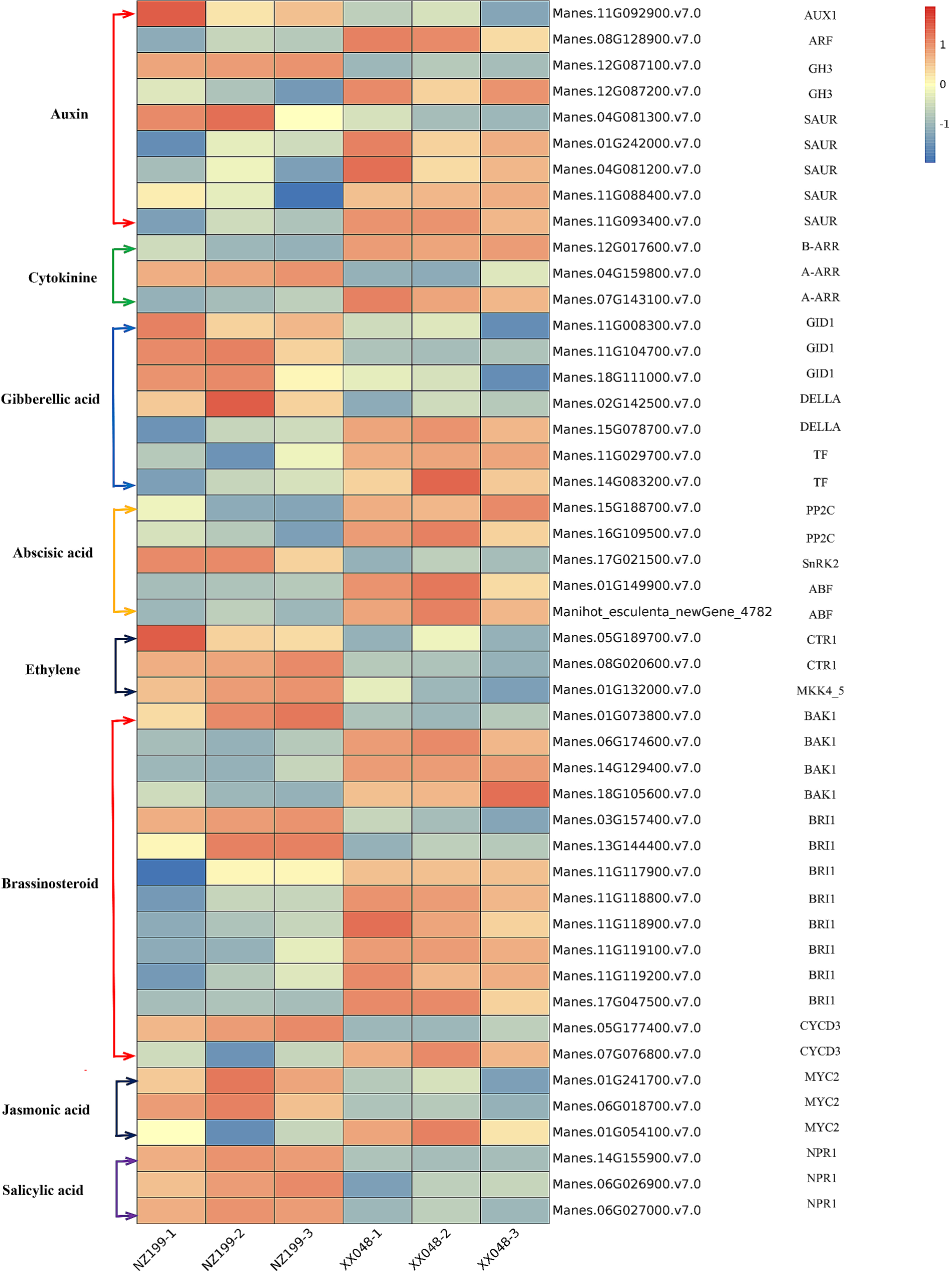
Heatmaps showing the DEGs involved in hormone signal transduction during cassava plant height growth in XX048 and NZ199 cassava variants

Plant hormone biosynthesis and signal transduction include IAA, GA, cytokinin, BR, ABA, ethylene (ETH), jasmonic acid (JA), and salicylic acid (SA) pathways. Notably, various DEGs involved in IAA, cytokinin, GA, and BR biosynthesis and signal transduction were found to be significantly down-regulated in NZ199 dwarf cassava (Fig. 8, Supplementary Table 2). For example, in IAA biosynthesis and transduction, auxin response factor (ARF), auxin-responsive GH3 gene family (GH3), and small auxin-up RNA (SAUR) were typically down-regulated in NZ199 plants. Meanwhile, in the cytokinin pathway, two-component response regulators in ARR-A and ARR-B families were predominantly down-regulated in NZ199 cassava. In the GA pathway of NZ199 cassava, one DELLA protein was up-regulated and another was down-regulated, while phytochrome-interacting factor 3 (TF) was down-regulated and the GA receptor GID1 was up-regulated. Several BR biosynthesis and signal transduction genes were also down-regulated in NZ199 cassava, including brassinosteroid insensitive 1-associated receptor kinase 1 (BAK1), protein brassinosteroid insensitive 1 (BRI1), and cyclin D3 (CYCD3).

Conversely, many important DEGs involved in ETH, SA, JA, and ABA biosynthesis and signal transduction were significantly up-regulated in dwarf cassava. For example, in the ABA pathway, serine/threonine-protein kinase SRK2 (SnRK2) and ABA-responsive element binding factor (ABF) were typically up-regulated in NZ199 dwarf cassava. Additionally, in the JA pathway, the transcription factor MYC2 was predominantly up-regulated in NZ199 plants. In the ETH and SA pathways, the serine/threonine-protein kinase CTR1, mitogen-activated protein kinase kinase 4/5 (MKK4/5), regulatory protein NPR1, and pathogenesis-related protein 1 (PR1) were up-regulated.

### DEGs associated with flavonoid biosynthesis and phenylpropanoid biosynthesis

**Fig. 9:**
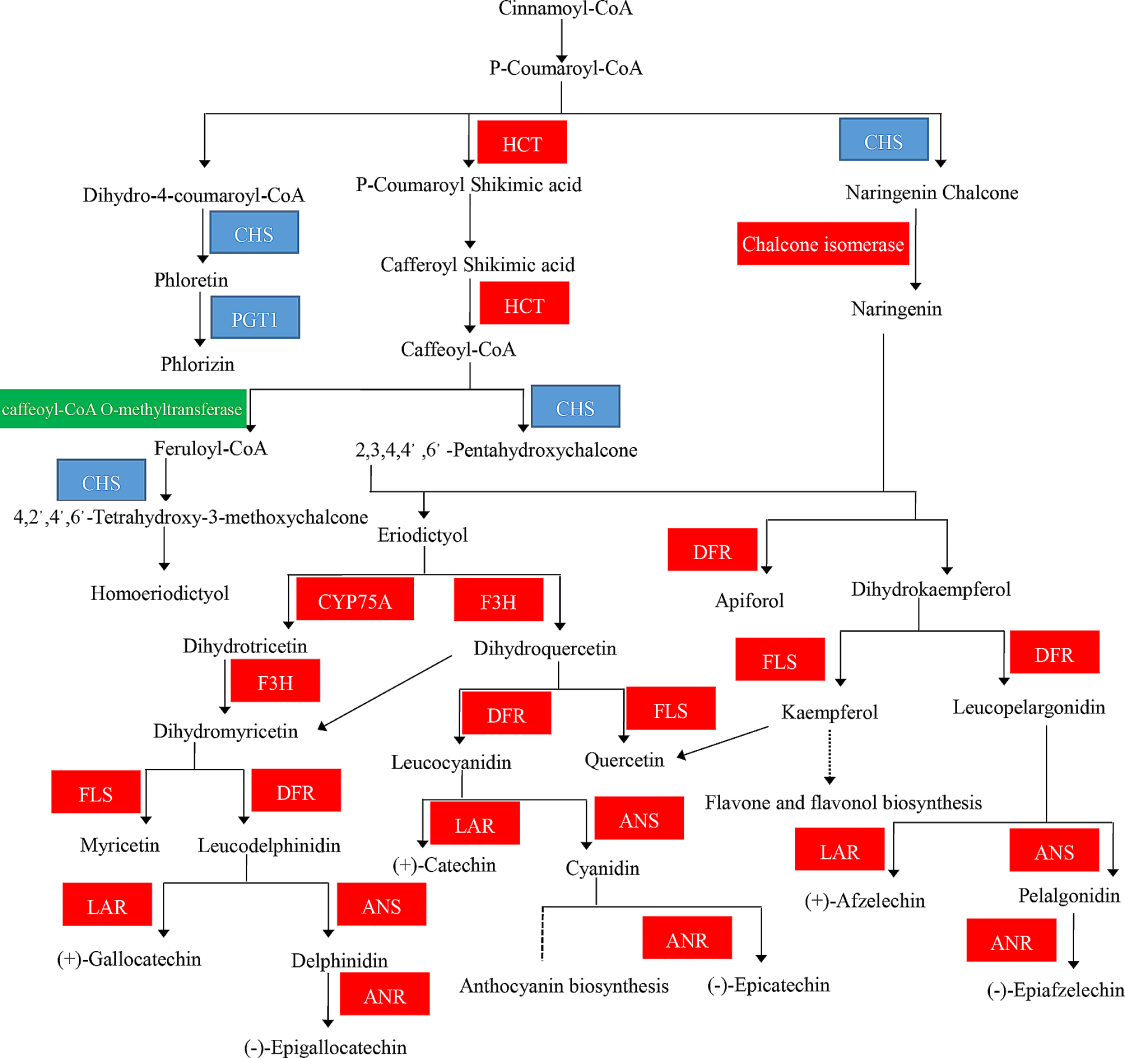
Mechanisms of flavonoid biosynthesis in cassava. HCT, shikimate O-hydroxycinnamoyl transferase; CHS, chalcone synthase; PGT1, phlorizin synthase; CYP75A, flavonoid 3’,5’-hydroxylase; F3H, naringenin 3-dioxygenase; DFR, bifunctional dihydroflavonol 4-reductase/flavanone 4-reductase; FLS, flavonol synthase; LAR, leucoanthocyanidin reductase; ANS, anthocyanidin synthase; ANR, anthocyanidin reductase. Red and green indicate up- and downregulated genes, respectively; blue indicates some upregulated and some downregulated genes

Overall, flavonoid biosynthesis was the most significantly enriched pathway in KEGG enrichment analysis. In this pathway, most associated genes were significantly up-regulated in the XX048 vs. NZ199 groups. Up-regulated DEGs included 4 chalcone synthase genes (CHS, EC: 2.3.1.74), 5 shikimate O-hydroxycinnamoyltransferase (HCT, EC: 2.3.1.133), 1 phlorizin synthase genes (PGT1, EC: 2.4.1.357), 2 chalcone isomerase (EC: 5.5.1.6), 1 naringenin 3-dioxygenase (F3H, EC: 1.14.11.9), 1 bifunctional dihydroflavonol 4-reductase/flavanone 4-reductase (DFR, EC: 1.1.1.219, EC: 1.1.1.234), 1 flavonoid 3’,5’-hydroxylase (CYP75A, EC: 1.14.14.81), 1 flavonol synthase (FLS, EC: 1.14.20.6), 1 anthocyanidin synthase (ANS, EC: 1.14.20.4), 1 anthocyanidin reductase (ANR, EC: 1.3.1.77), and 1 leucoanthocyanidin reductase (LAR, EC: 1.17.1.3). Meanwhile, only 1 chalcone synthase gene (CHS, EC: 2.3.1.74), 1 caffeoyl-CoA O-methyltransferase gene (EC: 2.1.1.104), and 5 phlorizin synthase genes (PGT1, EC: 2.4.1.357) were significantly down-regulated in the XX048 vs. NZ199 groups (Fig. 9, Supplementary Table 3).

**Fig. 10:**
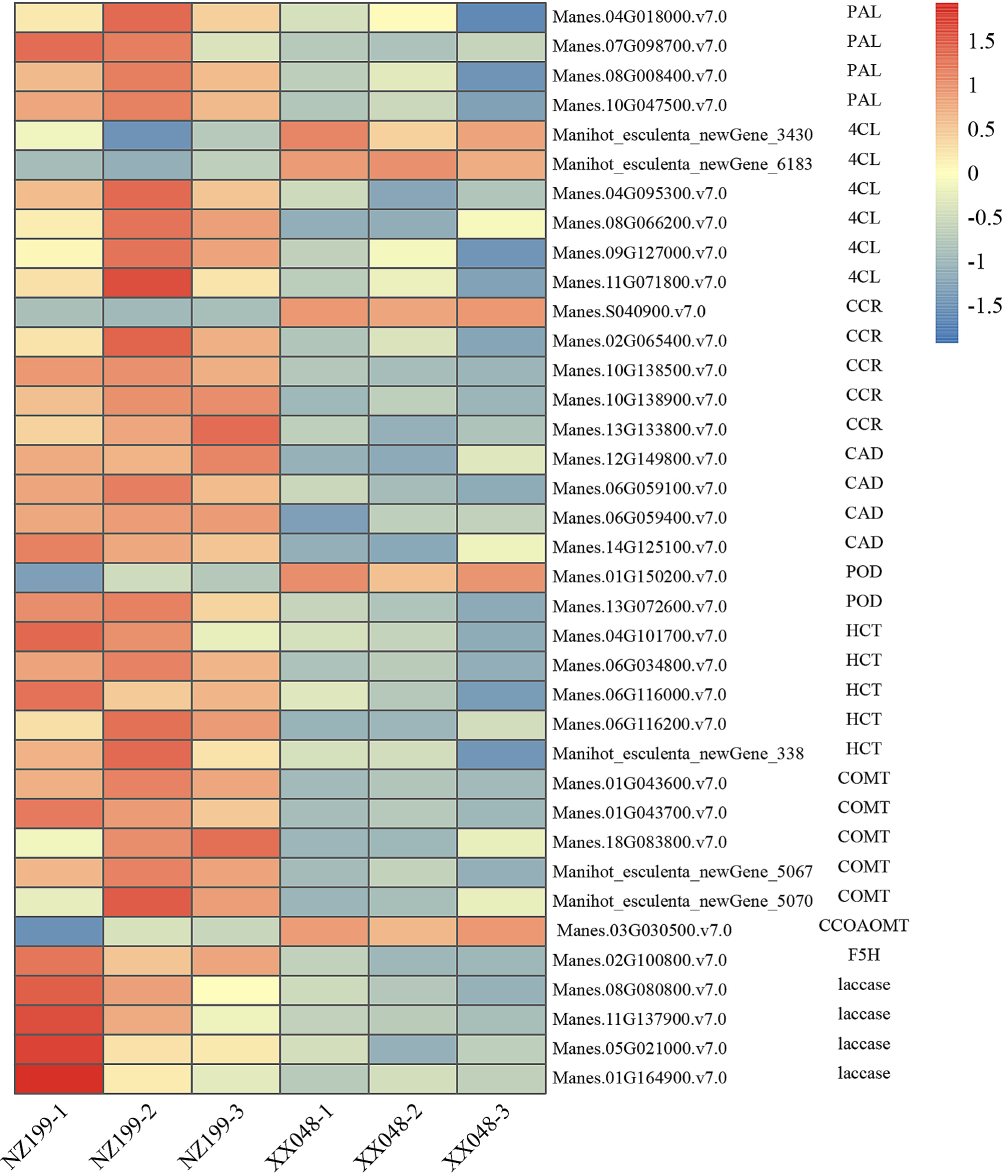
Heat maps depicting the differential expression patterns of genes involved in lignin biosynthesis in the XX048 and NZ199 cassava. PAL, phenylalanine ammonia-lyase; 4CL, 4-coumarate–CoA ligase; CCR, cinnamoyl-CoA reductase; CAD, cinnamyl-alcohol dehydrogenase; POD, peroxidase; HCT, shikimate O-hydroxycinnamoyltransferase; COMT, caffeic acid 3-O-methyltransferase/acetylserotonin O-methyltransferase; CCoAOMT, caffeoyl-CoA O-methyltransferase

Phenylpropanoid biosynthesis is another key pathway identified in KEGG enrichment analysis. The products of this pathway primarily include lignin monomers, including p-coumaroyl, coniferyl alcohol, and sinapyl alcohol. Notably, our study identified 37 DEGs involved in lignin monomer synthesis (Fig. 10, Supplementary Table 4), including 4 phenylalanine ammonia-lyase (PAL), 6 4-coumarate-CoA ligase (4CL), 5 cinnamoyl-CoA reductase (CCR), 4 cinnamyl-alcohol dehydrogenase (CAD), 2 peroxidases (POD), 5 HCT, 5 caffeic acid 3-O-methyltransferase/acetylserotonin O-methyltransferase (COMT), 1 caffeoyl-CoA O-methyltransferase (CCoAOMT), 1 ferulate-5-hydroxylase (F5H), and 4 laccase genes. Of these 37 DEGs, 28 were significantly up-regulated in the XX048 vs. NZ199 groups, while only 9 genes were significantly down-regulated.

### DEGs associated with cell wall biosynthesis and expansion

The synthesis of new cell wall components is essential to provide mechanical strength to newly elongated cell walls and internodes. In this study, we analyzed the differential expression of genes involved in cell wall synthesis or modification since they may be linked to cassava stem elongation and expansion; such genes include cellulose synthase (CES), EXP, xylosidase (XYL), pectin lyase (PL), pectin esterase (PM), pectin acetyl esterase (PA), and polygalacturonase (PG).

**Fig. 11:**
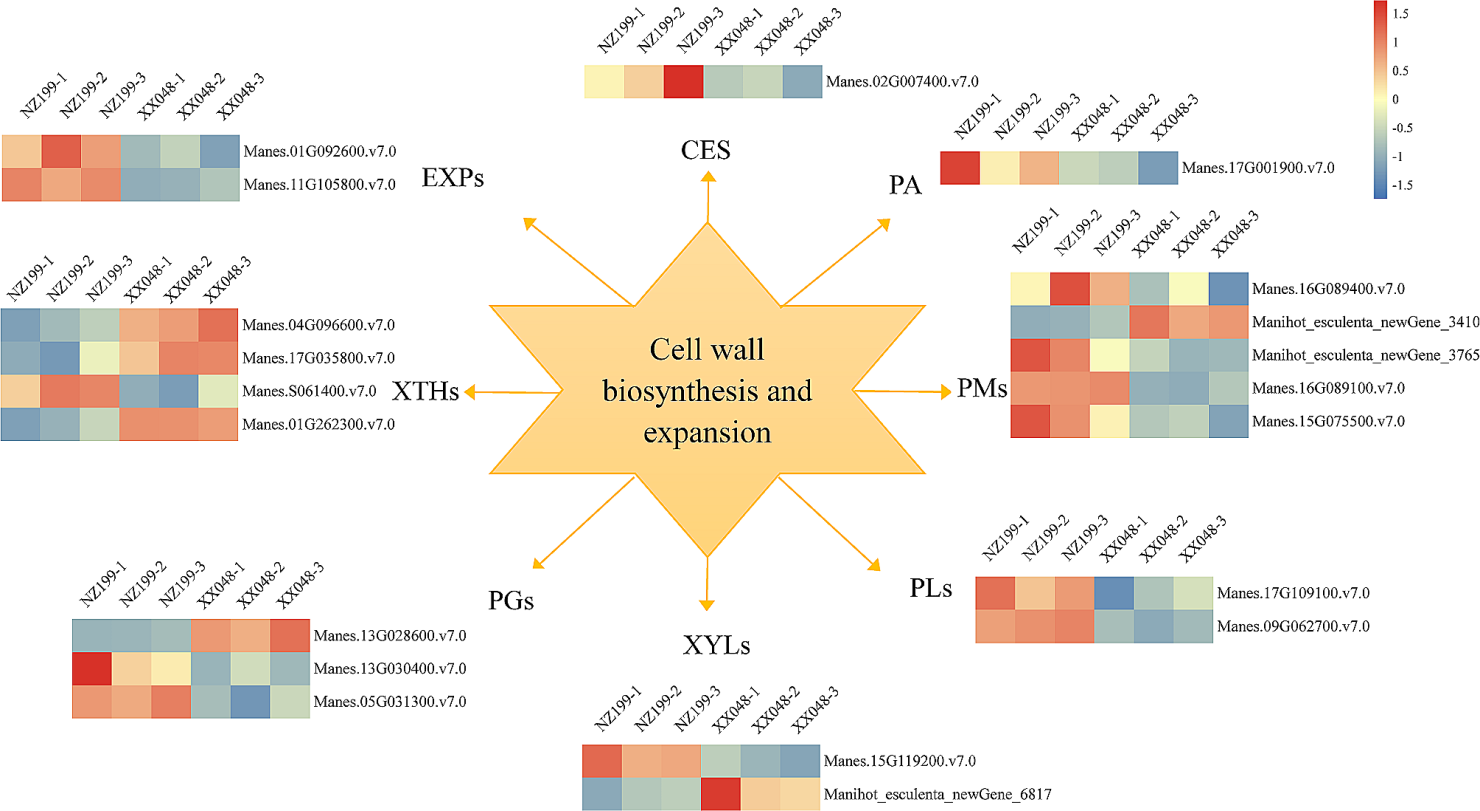
Heatmap showing the differential expression patterns of genes related to cell wall biosynthesis and expansion in the XX048 and NZ199 cassava variants. XTH, xyloglucan endotransglucosylase/hydrolase; CES, cellulose synthase; XYL, xylosidase; PL, pectate lyase; PM, pectinesterase; PA, pectin acetylesterase; PG, polygalacturonase; EXP, expansin

EXPs, nonhydrolytic cell wall-loosening proteins, enable cell expansion while facilitating differentiation and growth. In the present study, two members of the EXP gene family were found to be differentially expressed in tall and dwarf cassava, with significant up-regulation in NZ199 dwarf cassava (Fig. 11, Supplementary Table 5). Xyloglucan endoglycosyltransferase/hydrolase (XTH) also participates in cell expansion by promoting the relaxation and rearrangement of cell wall fibers in growing tissues. Notably, four XTHs were differentially expressed between tall and dwarf cassava, of which 3 were down-regulated and 1 was up-regulated in the tall vs. dwarf groups (Fig. 11, Supplementary Table 5). The degradation of pectin, a complex polymer, has been linked to the initiation of secondary wall lignification and cell wall disintegration. A total of 11 pectin-related genes were significantly differentially expressed between tall and dwarf cassava; this included 5 pectin esterase genes (PM), 3 polygalacturonase genes (PG), 2 pectin lyase genes (PL), and 1 pectin acetyl esterase gene (PA). Additionally, 1 cellulose synthase gene and 2 XYL genes were differentially expressed between the tall and dwarf cassava variants (Fig. 11, Supplementary Table 5).

### Transcription factor analysis

In this study, we identified 92 differentially expressed transcription factors between XX048 and NZ199 cassava, across 34 transcription factor families. Among these transcription factors, 56 were down-regulated and 36 were up-regulated (Supplementary Table 6). Notably, these transcription factors were primarily comprised of the bHLH (9), MYB (9), C2H2 (7), NAC (5), MYB-related (5), bZIP (5), AP2 / ERF-ERF (5), and WRKY (4) transcription factor families.

### qRT-PCR validation

**Fig. 12:**
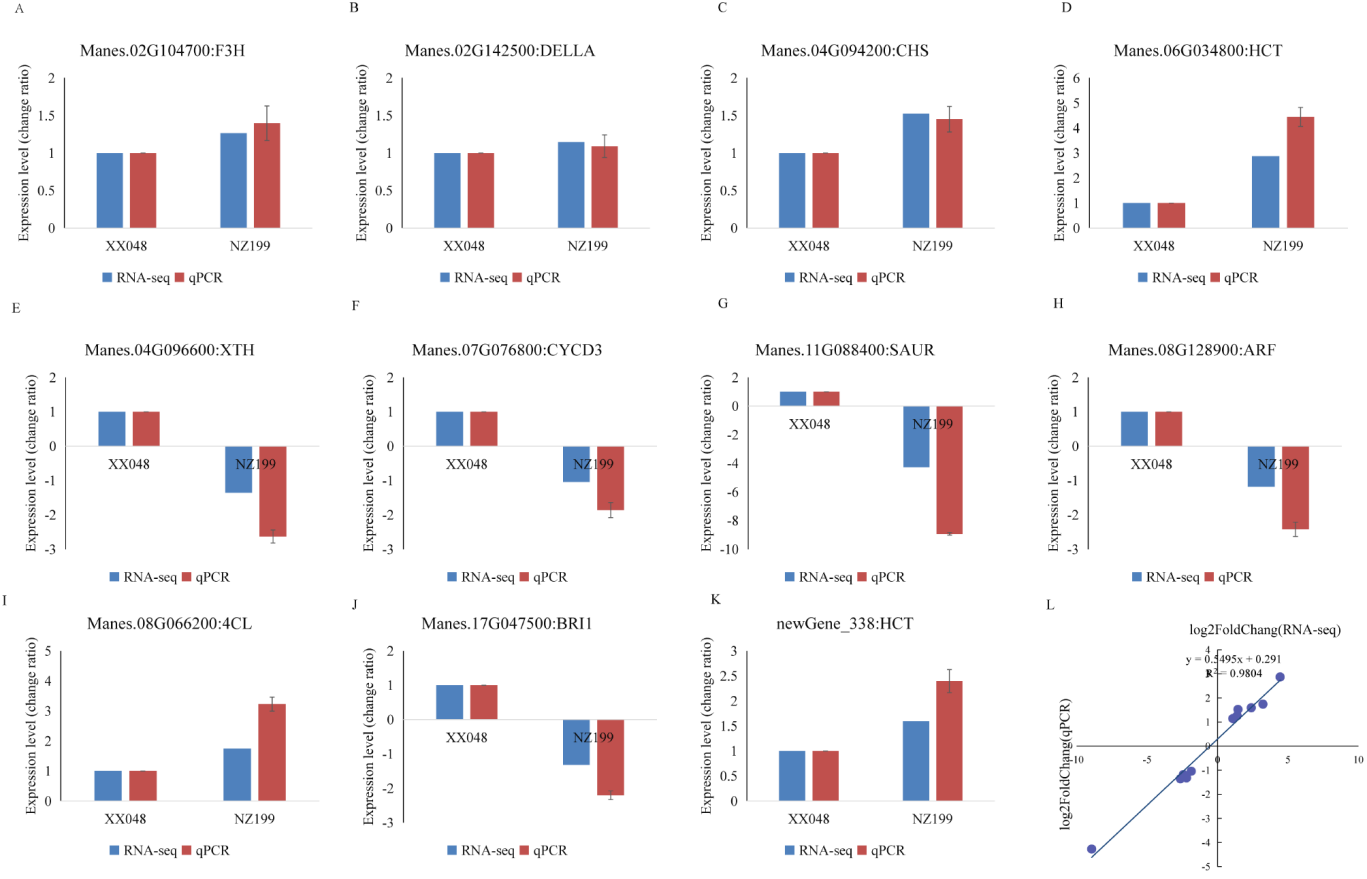
qRT-PCR validation of the transcriptome data. **a**-**k** qRT-PCR validation profles of eleven randomly selected genes. The data was normalized by using Actin as an internal reference. The expression level of XX048 was used as reference state, which was set to 1, and fold change values were shown here. **l** Pearson’s correlation analysis of the fold changes between qRT-PCR (x axis) and RNA-seq (y axis) data was conducted (R^2^)

To validate the accuracy of RNA-Seq results, 11 DEGs were randomly selected for qRT-PCR verification. Overall, the expression patterns of these 11 DEGs were similar to those identified by RNA-Seq, with an R^2^ value of 0.9804 (Fig. 12), indicating the reliability of our RNA-Seq results.

## Discussion

Plant height is an important agronomic trait affecting crop yield and quality. Therefore, understanding the molecular mechanism controlling crop plant height is of great significance in the agricultural sector. However, although cassava is considered one of three major potato crops and one of six major food crops globally, the genetic regulation mechanism of cassava plant height is unclear. Consequently, the regulation and genetic improvement of cassava plant height traits remain limited. Therefore, this study aimed to identify candidate genes and gene expression networks that are responsible for regulating cassava plant height development.

### Cell wall biosynthesis and expansion play a major role in plant height

Plant cell walls not only provide mechanical support for cell components and protects against various external stimuli, but is also essential for cell growth. Lignin and cellulose are key components of cell walls; therefore, their content is considered the primary factor affecting plant height. Notably, prior studies have established that lower lignin content is associated with increased plant height [30]. Changes in lignin content have been linked to the expression of genes related to lignin biosynthesis. Genes involved in lignin biosynthesis, such as PAL, HCT, 4CL, CCR, and CAD, play crucial regulatory roles, with lignin synthesis being positively correlated with the expression of these genes [31, 32]. In the present study, various PAL, HCT, 4CL, CCR, and CAD genes were found to be significantly up-regulated in dwarf cassava (Fig. 10), consistent with the observed physiological results indicating higher lignin content in dwarf cassava compared to tall cassava (Fig. 3). Similar results have also been observed in rice, with the stems of dwarf rice mutants displaying higher lignin content and higher lignin-related gene expression levels [33].

POD and laccase are also key components involved in lignin biosynthesis. Specifically, these enzymes catalyze cross-linking between phenolic groups in cell wall structural proteins, pectin, and other cell wall polymers, thereby inducing cell wall hardening, inhibiting cell elongation, and facilitating cell wall lignification. Notably, the present study revealed significant up-regulation in POD and laccase gene expression in dwarf cassava (Fig. 10). Ultimately, these results demonstrated that the cell wall lignification development of the two cassava varieties differed. In particular, the dwarf cassava entered the secondary wall lignification development state relatively earlier, resulting in the termination of cell elongation and the development of a dwarf phenotype.

Expansion is a process involving non-hydrolytic cell wall relaxation of protein in plant cell walls. It is considered to play an important role in regulating cell wall ductility and is a key factor in determining cell expansion [22]. The role of EXPs has been confirmed in various plant species. For example, overexpression of *OsEXP4* in transgenic rice can significantly increase plant height [23]. Similarly, overexpression of *ClEXPA2* in tobacco also significantly increases internode length [34]. However, although EXPs have long been thought to play an active role in promoting plant height and tissue expansion, this is not always the case. For example, Rochange et al. demonstrated that EXP overexpression in transgenic plants resulted in the development of smaller leaves and shorter internodes due to the reduced sensitivity of the cell wall to EXP [35]. Notably, a similar phenomenon was also observed in the current study. Specifically, two EXP genes were significantly up-regulated in dwarf cassava, indicating the normal transcription of EXPs (Fig. 11). Therefore, we hypothesize that a decrease in cell wall sensitivity to EXP is one of the primary factors affecting plant height dwarfing in cassava.

The XTH gene encodes a cell wall modifying enzyme that is found in higher plant cells; specifically, this enzyme alters cell wall structure by catalyzing the hydrolysis or transfer of xyloglucan molecules. Prior studies have revealed that XTH expression is closely related to plant height development. For example, *OsXTH8* expression is positively correlated with plant height in rice [36]. Meanwhile, mutations in *AtXTH9* have been found to result in shorter internodes length in *Arabidopsis* spp. [37]. Similarly, our results demonstrated that the expression level of XTH genes was significantly higher in high-stem cassava than that in dwarf-stem cassava (Fig. 11), indicating that these genes may be involved in cassava plant height development.

### Role of plant hormones in plant height development

As a physiological signal regulating plant growth and development, plant hormones play an important role in plant morphogenesis, growth, and metabolism. Notably, previous studies have revealed that dwarfing mutations are closely associated with plant hormones.

IAA, a key growth regulator, plays an important role in all stages of plant growth and development. Thus, abnormal expression of IAA transport key genes can lead to plant dwarfing. In the current study, the IAA response gene CH3 (Manes.12G087200.v7.0) was found to be significantly down-regulated in dwarf cassava, indicating a reduction in the IAA response. ARF transcription factors also mediate IAA responses and modulate stem development by regulating cell division [38]. In the present study, ARF expression was significantly down-regulated in dwarf cassava (Fig. 8), indicating inhibition of cell division. SAUR, the largest family of IAA response genes, also plays a key role in dwarfing; notably the gain-of-function of SAUR19–24 in seedlings has been found to result in increased hypocotyl length and leaf size, while the loss-of-function of lead to reduced hypocotyl length and leaf size [39]. Similarly, in the present study, a large number of SAUR genes were significantly down-regulated in dwarf cassava (Fig. 8); similar results have been observed in *Sophora davidii* and peanut plants [40, 41]. Overall, our findings indicate that these genes influence plant height during cassava growth and development.

Notably, GAs also significantly influence plant stem elongation. The plant hormone GA, the receptor GID1, and the inhibitor DELLA collectively represent the GA signal transduction pathway When activated by GA, the GID1 receptor binds to DELLA through the E3 ubiquitination pathway, leading to DELLA protein degradation and the obstruction of DELLA-mediated growth inhibition [42]. Thus, a loss-of-function mutation in the GID1 gene has been found to result in a dwarf phenotype [43]. Conversely, in the present study, GID1 was significantly up-regulated in dwarf cassava (Fig. 8). This contrasts with the understanding that loss-of-function mutations in GID1 cause plants to exhibit dwarf phenotypes and GA insensitivity [42]. Nonetheless, our finding aligns with the established GA-triggered negative feedback loop observed in in *Lepidium sativum* that results in the repression of GID1 gene transcription [44]. Similar results have been observed in studies on bamboo. For example, higher expression levels of *PeGID1* have been detected in the internodes of dwarf Moso bamboo than in wild-type bamboo [45]. DELLA is a transcription regulatory factor that responds to GA and acts as an inhibitory factor for GA-induced growth. In a prior study, the DELLA protein gene *PhSLR1* in moso bamboo was transferred into *Arabidopsis thaliana*; notably, these transgenic plants were found to be shorter than their wild-type counterparts [46]. In the present study, our findings aligned with those of previous studies, with one DELLA protein gene being up-regulated in dwarf cassava (Fig. 8). Therefore, cassava plant height dwarfing may be attributed to GA signal transduction inhibition by DELLA protein.

BRs are crucial positive regulators of stem elongation. Thus, the deletion or mutation of genes, transcription factors, and enzymes related to BR synthesis and signal transduction pathways reduce plant cell elongation, resulting in dwarfing. BRI1 is the primary component involved in BR signal detection; therefore, mutations in the BRI1 gene in *Arabidopsis* and rice spp. result in the development of dwarfing phenotypes [47–49]. Additionally, BAK1 also plays a key role in BR signal transduction. Consequently, BAK1 loss-of-function mutations have been shown to reduced BR sensitivity, leading to the development of shorter stems [50]. Meanwhile, inhibition of CYCD3, a D-type plant cyclin gene that promotes plant cell division, has been found to result in significant dwarfing in *Arabidopsis* spp. [51]. In the present study, the expression levels of 6 BRI1s, 3 BAK1s, and 1 CYCD3 genes were down-regulated in dwarf cassava (Fig. 8), indicating a reduction in BR sensitivity in dwarf cassava; therefore, the inhibition of cell elongation may serve as an important factor in cassava dwarfing.

### Role of flavonoids in plant height development

In recent years, many studies have shown the relationship between plant height development is related to flavonoids. Transcriptome and metabolome analysis has identified a series of genes involved in the flavonoid biosynthesis pathway that are differentially expressed in dwarf and tall mutants of *Sophora davidii*. A total of 8 differential metabolites were identified between these mutants, of which 6 were flavonoids, indicating that flavonoid metabolites play an important role in plant height morphogenesis in *S. davidii* [40]. Similarly, the dwarfing mechanism of Polish dwarf wheat was identified via transcriptome and proteome analysis; these results demonstrated that genes involved in flavonoid synthesis are up-regulated in dwarf wheat, resulting in increased flavonoid content, which limits IAA transport and leads to dwarfing [52]. Overall, these findings demonstrate that flavonoids regulate plant height development by controlling the polar transport of IAA. Similar to results of previous studies, our study revealed that the flavonoid biosynthesis was the most significantly enriched pathway in cassava; in particular, a series of genes involved in the flavonoid biosynthesis pathway (F3H, CHS, FLS, HCT, and F3′5′H) were found to be significantly up-regulated in dwarf cassava (Fig. 9). The up-regulated expression of these genes increases the flavonoid content of dwarf cassava, thereby inhibiting the polar transport of IAA; this inhibited IAA transport reduces cell ductility and ultimately limits cell expansion, resulting in dwarfing. However, the exact mechanisms involved in this process require further investigation.

### Role of transcription factors in plant height development

Some transcription factors, such as GRF, PRE, bZIP, TCP, and WRKY, have been shown to play important roles in plant height development. In the present study, we identified a total of 92 transcription factors differentially expressed between XX048 and NZ199 cassava, of which bHLH and MYB genes were the predominant transcription factor families.

bHLHs regulate various growth and development processes, including seed germination, floral organ development, and stem and lateral root growth. In *Arabidopsis* spp., the mutation of bHLH transcription factor SPATULA has been found to limit cell proliferation and expansion, resulting in shortened internode length [53, 54]. Meanwhile, maize double mutants (bHLH12/14) exhibit early internode differentiation and abnormal branching, resulting in dwarfism and reduced stem veins [55]. In the present study, most bHLH transcription factors were significantly down-regulated in dwarf cassava; ultimately, this may have resulted in the inhibition of cell proliferation and expansion.

MYB transcription factors are a large class of plant transcription factors involved in physiological and biochemical processes, including root development, cell development, secondary cell wall synthesis, and seed development. In rice, overexpression of the MYB transcription factor OsMPH1 increases plant height by increasing the length of internode cells [56]. MYB transcription factors are also involved in plant hormone signal transduction. For example, MYB33 positively regulates GA signal transduction during plant growth [57]. bZIP is also involved in plant height growth and development. In *Arabidopsis* spp., the bZIP transcription factor influences plant height growth and development by controlling GA activity [58, 59]. In the present study, most MYB and bZIP transcription factors were significantly down-regulated in dwarf cassava. Ultimately, these genes may be involved in GA signal transduction in cassava, serving as positive regulators of plant height development. However, the regulatory effects of these transcription factors on cassava plant height development requires further verification.

### Regulatory networks associated with cassava plant height development

**Fig. 13:**
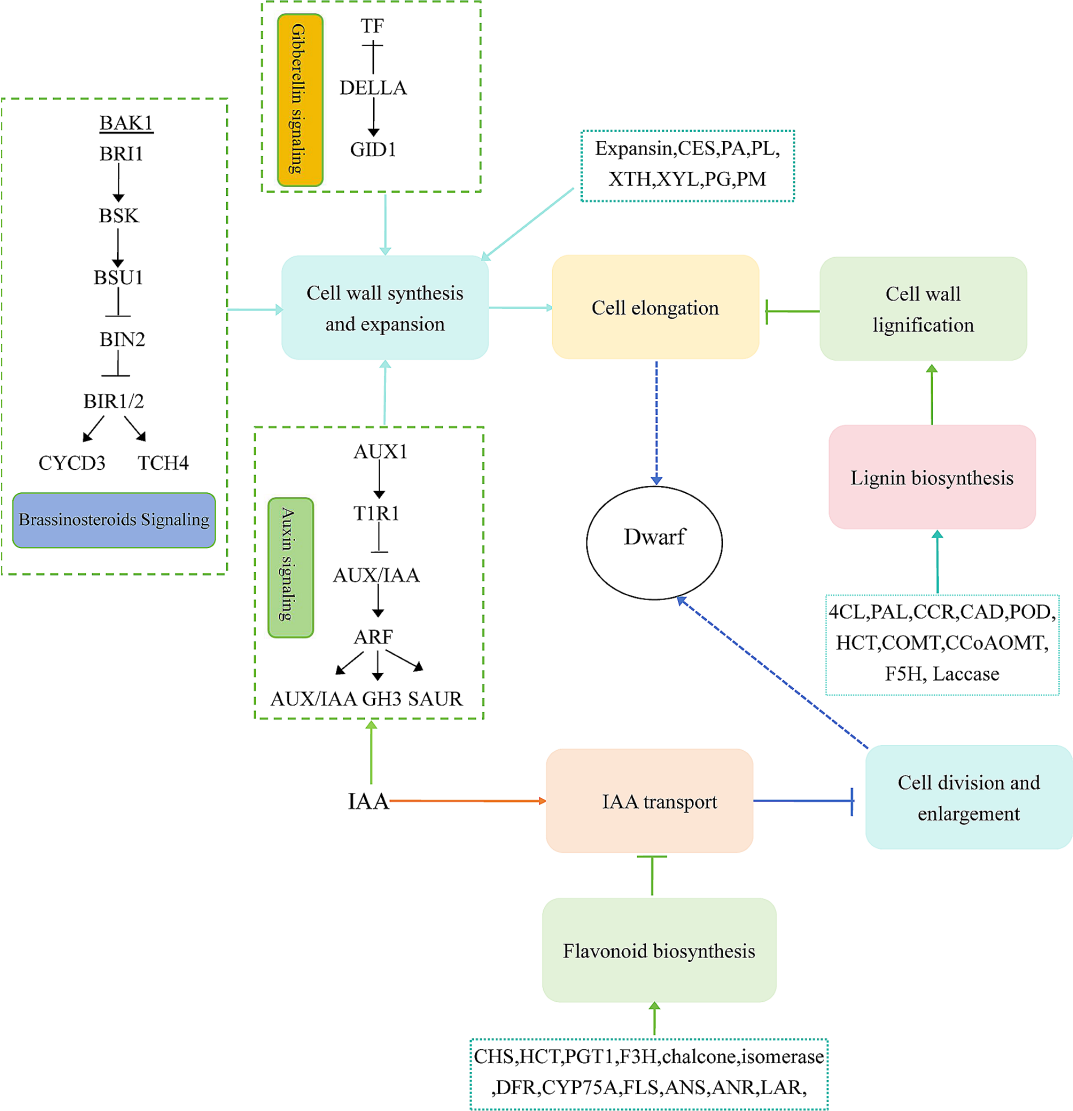
A hypothetical model illustrating the regulatory network related to plant height growth in cassava

Cassava plant height development is a complex regulatory process influenced by various factors. Based on the results of this study and previous research, we proposed a regulatory network model related to cassava plant height development (Fig. 13). Notably, increased cell wall lignification, reduced expression of plant hormone signal transduction–related genes, and excessive accumulation of flavonoid compounds are key factors involved in cassava plant height dwarfing. Furthermore, down-regulation of genes involved in cell wall synthesis and expansion, alongside downregulation of related transcriptional regulatory factors, may also influence height development in cassava. Specifically, the expression of genes related to the hormone signaling pathway and cell wall synthesis is down-regulated in dwarf cassava, resulting in the inhibition of cell wall biosynthesis and expansion. Additionally, some transcription factors influence cell development by participating in plant hormone signal transduction. The up-regulated expression of certain enzymes involved in lignin synthesis promotes cell wall lignification, thereby preventing cell elongation and growth and leading to dwarfing. Meanwhile, the up-regulated expression of flavonoid biosynthesis–related genes promotes the accumulation of flavonoids; these compounds inhibit IAA transport, which inhibits cell division and expansion and results in plant height dwarfing. In summary, the DEGs identified in this study play an important role in the regulatory network of cassava plant height development. Nonetheless, further functional identification studies are needed to elucidate the function of these candidate genes.

## Conclusion

This study analyzed transcriptome, physiological, and anatomical data to identify candidate genes related to cassava plant height development, ultimately proposing a genetic regulatory network model for cassava plant height development based on these results. Overall, we revealed the molecular mechanisms underlying the influence of plant hormone signaling, transcription factors, cell wall synthesis and elongation, flavonoid biosynthesis, and cell wall lignification on cassava plant height development. Therefore, our study provides a theoretical basis for understanding the molecular regulatory mechanisms of cassava plant height development; ultimately, these insights can be utilized to improve cassava crop traits via genetic breeding.

## Materials and methods

### Plant materials, growth conditions, and measurements of agronomic traits

In this study, the industrial cassava variants Xinxuan048 (XX048: high-stem) and Nanzhi199 (NZ199: dwarf-stem) were used. All plants were grown at the test base of the Guangxi South Asia Tropical Agricultural Science Research Institute, Longzhou County, Chongzuo City, China, under similar conditions. This test base, situated at an altitude of 138 m, experiences a southern subtropical monsoon climate, with an average annual temperature ≥ 23.1 °C, annual rainfall ≥ 1266 mm, annual sunshine of approximately 1521 h, an average annual relative humidity of approximately 88%, and an annual frost-free period of 361 days. The test base consisted of loam soil with medium fertility. The cassava were planted in April 2022 using a completely randomized block design, with each variety treated as a single treatment and repeated three times. The planting distance was 1 m between rows and 0.8 m between individual plants. Field management followed standard agricultural practices.

Two cassava lines, XX048 and NZ199, with uniform growth were selected to evaluate plant height during the stem growth period. The topmost internode tissue was collected for cytological observation [40] when the plant height difference between XX048 and NZ199 was the largest (September 2022, during the tuberous root expansion period). The microstructure of XX048 and NZ199 cassava was then compared, and the content of cellulose and lignin, the primary components of the cell wall was measured. Transcriptome sequencing was also conducted for further comparison.

### Cytological observation

In September 2022, 5 plants with consistent growth were randomly selected from each planting plot. The topmost internodes tissues from XX048 and NZ199 were collected and named XX048-1, XX048-2, XX048-3, NZ199-1, NZ199-2 and NZ199-3. These samples were immediately fixed in formalin-aceto-alcohol for over 24 h. Before dehydration, plant lignocellulose was softened with an acidic softening solution for several days to a week. The samples were then dehydrated with gradient ethanol (75%, 85%, 90%, 95%, 100%, 100%) sequentially (4 h, 2 h, 2 h, 2 h, 2 h, 2 h, 2 h), followed by infiltration with alcohol benzene, transparent agent I, and transparent agent II for 30 min each, before performing paraffin embedding. Subsequently, 8 μm thick serial sections were cut using a 340E microtome (ThermoFisher Scientific, China) and fixed on slides. Finally, the sections were stained with toluidine blue, dehydrated, made transparent, sealed with neutral gum, and observed with an Olympus BX53F2 photomicroscope (Olympus, Tokyo, Japan).

### Determination of lignin and cellulose content

In September 2022, 5 plants with consistent growth were randomly selected from each planting plot. The topmost internodes tissues from XX048 and NZ199 were then collected. The collected samples were dried at 80 °C to a constant weight, crushed, and sieved through 40 mesh. Subsequently. lignin and cellulose contents were determined using a kit from Suzhou Keming Biotechnology Co. Ltd., (China), following the manufacturer’s instructions.

### Sample collection, RNA extraction, RNA sequencing, and data analysis

In September 2022, 5 plants with consistent growth were randomly selected from each planting plot. The topmost internodes tissues from XX048 and NZ199 were then collected and stored at − 80 °C for total RNA extraction.

Total RNA was extracted using the RNAprep Pure Plant Kit (Tiangen, Beijing, China), according to the manufacturer’s instructions. RNA concentration and purity were measured using a NanoDrop 2000 spectrophotometer (Thermo Fisher Scientific, Wilmington, DE). Meanwhile, RNA integrity was assessed using the RNA Nano 6000 Assay Kit with the Agilent Bioanalyzer 2100 system (Agilent Technologies, CA, USA).

For RNA sample preparations, 1 µg of RNA per sample was used as the input material. Sequencing libraries were then generated using the Hieff NGS Ultima Dual-mode mRNA Library Prep Kit for Illumina (Yeasen Biotechnology Co., Ltd., Shanghai, China), following the manufacturer’s recommendations; index codes were added to attribute sequences to each sample. Briefly, mRNA was purified from total RNA samples using poly-T oligo-attached magnetic beads. Subsequently, first-strand and second-strand cDNA synthesis were performed. Blunt ends were then formed using exonuclease and polymerase enzymes. After adenylation of 3′ ends, the NEBNext Adaptor with a hairpin loop structure was ligated to prepare for hybridization. The library fragments were purified using the AMPure XP system (Beckman Coulter, Beverly, USA). Then, 3 µl of USER Enzyme (NEB, USA) was used to treat size-selected, adaptor-ligated cDNA at 37℃ for 15 min, followed by 5 min at 95℃. Subsequently, PCR was performed with Phusion High-Fidelity DNA polymerase, universal PCR primers, and Index (X) Primer. PCR products were purified using the AMPure XP system and library quality was assessed with the Agilent Bioanalyzer 2100 system. Clean reads were obtained by removing reads containing adapters, ploy-N sequences, and low-quality reads from raw data. Q20, Q30, GC-content, and sequence duplication levels of the clean reads were calculated. All subsequent analyses were based on the high-quality clean data, including mapping to the cassava genome sequence. Gene expression levels were estimated as fragments per kilobase of transcript per million fragments mapped. DEGs were identified with an adjusted P-value < 0.01 and a log2 fold change > 1 using DESeq2. Finally, gene functions were annotated based on the following databases: NR (NCBI non-redundant protein sequences), Pfam (Protein family), KOG/COG (Clusters of Orthologous Groups of proteins), Swiss-Prot (A manually annotated and reviewed protein sequence database), KO (KEGG Ortholog database), and GO (Gene Ontology).

### Fluorescence quantitative verification

Real-time quantitative polymerase chain reaction (qPCR) was performed as described by Cai et al. [60]. The primer sequences used in this study are shown in Supplementary Table S7. Relative expression levels were calculated using the 2^−ΔΔCT^ method with actin as the endogenous reference gene.

### Statistical analysis

Plant height data were collected and processed using Excel 2010 software. The statistical analysis of all differences between the means was performed by using ANONA and DUNCAN with the IBM SPSS statistics 20 program. Image-Pro Plus 6.0 software was used for data analysis of the slices, and Excel 2010 was used for mapping. All heatmapping was done using the OECloud tool on https://cloud.oebiotech.com.

### Electronic supplementary material

Below is the link to the electronic supplementary material.

Supplementary Material 1

## Data Availability

The raw sequencing data have been submitted to the NCBI SRA database (PRJNA976406).

## Declarations

### Method declaration

All methods used in this study were implemented in accordance with relevant guidelines.

## Abbreviations

BR: Brassinosteroid
GA: Gibberellin
IAA: Auxin
SL: Strigolactone
SA: Salicylic acid
JA: Jasmonic acid
ABA: Abscisic acid
XTH: Xyloglucan endotransglucosylase/hydrolases
CesA: Cellulose synthase
CSL: Cellulose synthase-like
CYCD3: Cyclin D3
SnRK2: Serine/threonine-protein kinase SRK2
ETH: Ethylene
CHS: Chalcone synthase
HCT: Shikimate O-hydroxycinnamoyltransferase
PGT1: Phlorizin synthase
F3H: Naringenin 3-dioxygenase
DFR: Bifunctional dihydroflavonol 4-reductase/flavanone 4-reductase
CYP75A: Flavonoid 3’,5’-hydroxylase
ANS: Anthocyanidin synthase
ANR: Anthocyanidin reductase
LAR: Leucoanthocyanidin reductase
PAL: Phenylalanine ammonia-lyase
4CL: 4-coumarate–CoA ligase
CCR: Cinnamoyl-CoA reductase
CAD: Cinnamyl-alcohol dehydrogenase
POD: Peroxidase
HCT: Shikimate O-hydroxycinnamoyltransferase
COMT: Caffeic acid 3-O-methyltransferase/acetylserotonin O-methyltransferase
CCoAOMT: Caffeoyl-CoA O-methyltransferase
CES: Cellulose synthase
EXP: Expansin
XYL: Xylosidase
PL: Pectin lyase
PM: Pectin esterase
PA: Pectin acetyl esterase
PG: Polygalacturonase
XTH: Xyloglucan endoglycosyltransferase/hydrolase
