# Supplementary material for: Morphological, anatomical, and transcriptomics analysis reveals the regulatory mechanisms of cassava plant height development
Source: BMC Genomics. 2024 Jul 17;25:699. doi: 10.1186/s12864-024-10599-2 (PMC11253480; doi:10.1186/s12864-024-10599-2)
Supplement: Supplementary file 1 — Supplementary Material 1 [file 12864_2024_10599_MOESM1_ESM.docx]

**Supplementary data**

**Table S1** Quality statistics of original sequencing data and alignment analysis of filtered data with reference genome sequence

| Sample | NZ199-1 | NZ199-2 | NZ199-3 | XX048-1 | XX048-2 | XX048-3 |
| --- | --- | --- | --- | --- | --- | --- |
| Clean reads | 21685438 | 20588117 | 19435081 | 19099306 | 20629701 | 20353808 |
| Clean bases | 6494030802 | 6164022232 | 5817769286 | 5717066602 | 6175760156 | 6092129602 |
| Q20(%) | 98.07 | 97.85 | 97.87 | 97.97 | 97.93 | 97.82 |
| Q30(%) | 94.14 | 93.69 | 93.76 | 94 | 93.89 | 93.65 |
| GC(%) | 43.19 | 43.02 | 42.87 | 42.35 | 43.41 | 42.74 |
| Total_map | 41,502,875 (95.69%) | 39,189,528 (95.18%) | 36,881,622 (94.88%) | 36,120,633 (94.56%) | 39,079,681 (94.72%) | 38,620,104 (94.87%) |
| Unique_map | 39,262,053 (90.53%) | 37,038,441 (89.95%) | 34,885,856 (89.75%) | 34,125,115 (89.34%) | 34,862,871 (84.50%) | 36,417,923 (89.46%) |
| Multi_map | 2,240,822 (5.17%) | 2,151,087 (5.22%) | 1,995,766 (5.13%) | 1,995,518 (5.22%) | 4,216,810 (10.22%) | 2,202,181 (5.41%) |

**Table S2** Quality statistics of original sequencing data and alignment analysis of filtered data with reference genome sequence

| Phythormone signal transduction pathways | Gene ID | Gene name | XX048 vs NZ199 | Description |
| --- | --- | --- | --- | --- |
| Auxin | Manes.11G092900.v7.0 | AUX1 | 1.934091941 | auxin influx carrier (AUX1 LAX family) |
|  | Manes.08G128900.v7.0 | ARF | -1.180964044 | auxin response factor |
|  | Manes.12G087100.v7.0 | GH3 | 1.12137177 | auxin responsive GH3 gene family |
|  | Manes.12G087200.v7.0 | GH3 | -1.121807248 | auxin responsive GH3 gene family |
|  | Manes.04G081300.v7.0 | SAUR | 1.651486438 | SAUR family protein |
|  | Manes.01G242000.v7.0 | SAUR | -1.32976518 | SAUR family protein |
|  | Manes.04G081200.v7.0 | SAUR | -1.971520249 | SAUR family protein |
|  | Manes.11G088400.v7.0 | SAUR | -4.273151417 | SAUR family protein |
|  | Manes.11G093400.v7.0 | SAUR | -1.027404003 | SAUR family protein |
|  |  |  |  |  |
| Cytokinin | Manes.12G017600.v7.0 | B-ARR | -3.823761742 | two-component response regulator ARR-B family |
|  | Manes.04G159800.v7.0 | A-ARR | 1.868657347 | two-component response regulator ARR-A family |
|  | Manes.07G143100.v7.0 | A-ARR | -2.413963646 | two-component response regulator ARR-A family |
|  |  |  |  |  |
| Gibberellic acid | Manes.11G008300.v7.0 | GID1 | 2.094528855 | gibberellin receptor GID1 |
|  | Manes.11G104700.v7.0 | GID1 | 1.139025571 | gibberellin receptor GID1 |
|  | Manes.18G111000.v7.0 | GID1 | 1.216190181 | gibberellin receptor GID1 |
|  | Manes.02G142500.v7.0 | DELLA | 1.144970127 | DELLA protein |
|  | Manes.15G078700.v7.0 | DELLA | -3.049885015 | DELLA protein |
|  | Manes.11G029700.v7.0 | TF | -1.526445428 | phytochrome-interacting factor 3 |
|  | Manes.14G083200.v7.0 | TF | -1.458895948 | phytochrome-interacting factor 3 |
|  |  |  |  |  |
| Abscisic acid | Manes.15G188700.v7.0 | PP2C | -1.681636172 | protein phosphatase 2C |
|  | Manes.16G109500.v7.0 | PP2C | -1.84112406 | protein phosphatase 2C |
|  | Manes.17G021500.v7.0 | SnRK2 | 2.458572883 | serine/threonine-protein kinase SRK2 |
|  | Manes.01G149900.v7.0 | ABF | -1.428334874 | ABA responsive element binding factor |
|  | Manihot_esculenta_newGene_4782 | ABF | -1.619859554 | ABA responsive element binding factor |
|  |  |  |  |  |
| Ethylene | Manes.05G189700.v7.0 | CTR1 | 1.965576331 | serine/threonine-protein kinase CTR1 |
|  | Manes.08G020600.v7.0 | CTR1 | 1.334754461 | serine/threonine-protein kinase CTR1 |
|  | Manes.01G132000.v7.0 | MKK4_5 | 2.424288308 | mitogen-activated protein kinase kinase 4/5 |
|  |  |  |  |  |
| Brassinosteroid | Manes.01G073800.v7.0 | BAK1 | 1.323776006 | brassinosteroid insensitive 1-associated receptor kinase 1 |
|  | Manes.06G174600.v7.0 | BAK1 | -1.008663081 | brassinosteroid insensitive 1-associated receptor kinase 1 |
|  | Manes.14G129400.v7.0; | BAK1 | -1.581696095 | brassinosteroid insensitive 1-associated receptor kinase 1 |
|  | Manes.18G105600.v7.0 | BAK1 | -1.209802956 | brassinosteroid insensitive 1-associated receptor kinase 1 |
|  | Manes.03G157400.v7.0 | BRI1 | 3.04383408 | protein brassinosteroid insensitive 1 |
|  | Manes.13G144400.v7.0 | BRI1 | 1.111484684 | protein brassinosteroid insensitive 1 |
|  | Manes.11G117900.v7.0 | BRI1 | -3.836759895 | protein brassinosteroid insensitive 1 |
|  | Manes.11G118800.v7.0 | BRI1 | -2.120685137 | protein brassinosteroid insensitive 1 |
|  | Manes.11G118900.v7.0 | BRI1 | -1.342318587 | protein brassinosteroid insensitive 1 |
|  | Manes.11G119100.v7.0 | BRI1 | -2.913272134 | protein brassinosteroid insensitive 1 |
|  | Manes.11G119200.v7.0 | BRI1 | -2.860830293 | protein brassinosteroid insensitive 1 |
|  | Manes.17G047500.v7.0 | BRI1 | -1.316607061 | protein brassinosteroid insensitive 1 |
|  | Manes.05G177400.v7.0 | CYCD3 | 1.268493795 | cyclin D3, plant |
|  | Manes.07G076800.v7.0 | CYCD3 | -1.04184009 | cyclin D3, plant |
|  |  |  |  |  |
| Jasmonic acid | Manes.01G241700.v7.0 | MYC2 | 2.085305006 | transcription factor MYC2 |
|  | Manes.06G018700.v7.0 | MYC2 | 1.295597435 | transcription factor MYC2 |
|  | Manes.01G054100.v7.0 | MYC2 | -1.391698595 | transcription factor MYC2 |
|  |  |  |  |  |
| Salicylic acid | Manes.06G026900.v7.0 | PR1 | 1.138750752 | pathogenesis-related protein 1 |
|  | Manes.06G027000.v7.0 | PR1 | 1.322132827 | pathogenesis-related protein 1 |

**Table S3**  DEGs involved in flavonoid biosynthesis

| Gene ID | Gene name | XX048 vs NZ199 | Description |
| --- | --- | --- | --- |
| Manes.11G089600.v7.0 | CHS | -9.63 | chalcone synthase |
| Manes.03G150000.v7.0 | CHS | 1.38 | chalcone synthase |
| Manes.04G094200.v7.0 | CHS | 1.53 | chalcone synthase |
| Manes.11G075100.v7.0 | CHS | 1.26 | chalcone synthase |
| Manes.11G075300.v7.0 | CHS | 1.34 | chalcone synthase |
| Manes.04G101700.v7.0 | HCT | 1.45 | shikimate O-hydroxycinnamoyltransferase |
| Manes.06G034800.v7.0 | HCT | 2.87 | shikimate O-hydroxycinnamoyltransferase |
| Manes.06G116000.v7.0 | HCT | 1.12 | shikimate O-hydroxycinnamoyltransferase |
| Manes.06G116200.v7.0 | HCT | 2.59 | shikimate O-hydroxycinnamoyltransferase |
| Manihot_esculenta_newGene_338 | HCT | 1.59 | shikimate O-hydroxycinnamoyltransferase |
| Manes.03G030500.v7.0 | EC:2.1.1.104 | -1.60 | caffeoyl-CoA O-methyltransferase |
| Manes.10G133600.v7.0 | PGT1 | -1.75 | phlorizin synthase |
| Manes.S036400.v7.0 | PGT1 | -2.37 | phlorizin synthase |
| Manes.S075200.v7.0 | PGT1 | -5.43 | phlorizin synthase |
| Manes.S111200.v7.0 | PGT1 | -2.85 | phlorizin synthase |
| Manihot_esculenta_newGene_454 | PGT1 | -2.07 | phlorizin synthase |
| Manes.14G020800.v7.0 | PGT1 | 1.24 | phlorizin synthase |
| Manes.07G107200.v7.0 | EC:5.5.1.6 | 1.48 | chalcone isomerase |
| Manes.16G058100.v7.0 | EC:5.5.1.6 | 1.70 | chalcone isomerase |
| Manes.02G104700.v7.0 | F3H | 1.26 | naringenin 3-dioxygenase |
| Manes.18G022300.v7.0 | DFR | 1.54 | bifunctional dihydroflavonol 4-reductase/flavanone 4-reductase |
| Manes.01G178000.v7.0 | CYP75A | 2.31 | flavonoid 3',5'-hydroxylase |
| Manes.02G160700.v7.0 | FLS | 1.10 | flavonol synthase |
| Manes.01G070200.v7.0 | ANS | 1.50 | anthocyanidin synthase |
| Manes.16G016400.v7.0 | ANR | 1.82 | anthocyanidin reductase |
| Manes.14G097300.v7.0 | LAR | 1.19 | leucoanthocyanidin reductase |

**Table S4** Relative changes in the expression of lignin biosynthesis-related genes

| Category | Gene ID | XX048 vs NZ199 | Description |
| --- | --- | --- | --- |
| PAL | Manes.04G018000.v7.0 | 1.21 | phenylalanine ammonia-lyase |
| PAL | Manes.07G098700.v7.0 | 2.31 | phenylalanine ammonia-lyase |
| PAL | Manes.08G008400.v7.0 | 1.27 | phenylalanine ammonia-lyase |
| PAL | Manes.10G047500.v7.0 | 1.56 | phenylalanine ammonia-lyase |
| 4CL | Manihot_esculenta_newGene_3430 | -1.09 | 4-coumarate--CoA ligase |
| 4CL | Manihot_esculenta_newGene_6183 | -1.94 | 4-coumarate--CoA ligase |
| 4CL | Manes.04G095300.v7.0 | 1.30 | 4-coumarate--CoA ligase |
| 4CL | Manes.08G066200.v7.0 | 1.74 | 4-coumarate--CoA ligase |
| 4CL | Manes.09G127000.v7.0 | 1.38 | 4-coumarate--CoA ligase |
| 4CL | Manes.11G071800.v7.0 | 1.27 | 4-coumarate--CoA ligase |
| CCR | Manes.S040900.v7.0 | -7.84 | cinnamoyl-CoA reductase |
| CCR | Manes.02G065400.v7.0 | 1.96 | cinnamoyl-CoA reductase |
| CCR | Manes.10G138500.v7.0 | 1.29 | cinnamoyl-CoA reductase |
| CCR | Manes.10G138900.v7.0 | 1.76 | cinnamoyl-CoA reductase |
| CCR | Manes.13G133800.v7.0 | 1.70 | cinnamoyl-CoA reductase |
| CAD | Manes.12G149800.v7.0 | 1.13 | cinnamyl-alcohol dehydrogenase |
| CAD | Manes.06G059100.v7.0 | 1.67 | cinnamyl-alcohol dehydrogenase |
| CAD | Manes.06G059400.v7.0 | 1.54 | cinnamyl-alcohol dehydrogenase |
| CAD | Manes.14G125100.v7.0 | 1.24 | cinnamyl-alcohol dehydrogenase |
| POD | Manes.01G150200.v7.0 | -1.00 | peroxidase |
| POD | Manes.13G072600.v7.0 | 1.67 | peroxidase |
| HCT | Manes.04G101700.v7.0 | 1.45 | shikimate O-hydroxycinnamoyltransferase |
| HCT | Manes.06G034800.v7.0 | 2.87 | shikimate O-hydroxycinnamoyltransferase |
| HCT | Manes.06G116000.v7.0 | 1.12 | shikimate O-hydroxycinnamoyltransferase |
| HCT | Manes.06G116200.v7.0 | 2.59 | shikimate O-hydroxycinnamoyltransferase |
| HCT | Manihot_esculenta_newGene_338 | 1.59 | shikimate O-hydroxycinnamoyltransferase |
| COMT | Manes.01G043600.v7.0 | 1.94 | caffeic acid 3-O-methyltransferase / acetylserotonin O-methyltransferase |
| COMT | Manes.01G043700.v7.0 | 1.55 | caffeic acid 3-O-methyltransferase / acetylserotonin O-methyltransferase |
| COMT | Manes.18G083800.v7.0 | 1.50 | caffeic acid 3-O-methyltransferase / acetylserotonin O-methyltransferase |
| COMT | Manihot_esculenta_newGene_5067 | 3.61 | caffeic acid 3-O-methyltransferase / acetylserotonin O-methyltransferase |
| COMT | Manihot_esculenta_newGene_5070 | 1.95 | caffeic acid 3-O-methyltransferase / acetylserotonin O-methyltransferase |
| CCoAOMT | Manes.03G030500.v7.0 | -1.60 | caffeoyl-CoA O-methyltransferase |
| F5H | Manes.02G100800.v7.0 | 2.48 | ferulate-5-hydroxylase |
| laccase | Manes.08G080800.v7.0 | 2.59 | Laccase-17 |
| laccase | Manes.11G137900.v7.0 | 2.24 | Laccase-17 |
| laccase | Manes.05G021000.v7.0 | 1.33 | Laccase-6 |
| laccase | Manes.01G164900.v7.0 | 2.35 | Laccase-4 |

**Table S5** Relative changes in expression of expansin-related genes

| Category | Gene ID | XX048 vs NZ199 | Description |
| --- | --- | --- | --- |
| CES | Manes.02G007400.v7.0 | 2.27 | Cellulose synthase-like protein G3 |
| expansin | Manes.01G092600.v7.0 | 2.76 | Expansin-A15 |
| expansin | Manes.11G105800.v7.0 | 1.51 | Expansin-like A2 |
| XTH | Manes.04G096600.v7.0 | -1.35 | Probable xyloglucan endotransglucosylase/hydrolase protein 5 |
| XTH | Manes.17G035800.v7.0 | -1.09 | Probable xyloglucan endotransglucosylase/hydrolase protein 8 |
| XTH | Manes.S061400.v7.0 | 1.58 | Probable xyloglucan endotransglucosylase/hydrolase protein 6 |
| XTH | Manes.01G262300.v7.0 | -1.87 | Probable xyloglucan endotransglucosylase/hydrolase protein 29 |
| XYL | Manes.15G119200.v7.0 | 2.22 | Probable beta-D-xylosidase 5 |
| XYL | Manihot_esculenta_newGene_6817 | -2.54 | Beta-xylosidase/alpha-L-arabinofuranosidase 2 |
| PL | Manes.17G109100.v7.0 | 1.25 | Pectate lyase |
| PL | Manes.09G062700.v7.0 | 3.98 | Probable pectate lyase 12 |
| PM | Manes.16G089400.v7.0 | 1.38 | Probable pectinesterase/pectinesterase inhibitor 54 |
| PM | Manihot_esculenta_newGene_3410 | -1.84 | Pectinesterase PPME1 |
| PM | Manihot_esculenta_newGene_3765 | 1.30 | Pectinesterase 2.1 |
| PM | Manes.16G089100.v7.0 | 2.02 | Pectinesterase/pectinesterase inhibitor PPE8B |
| PM | Manes.15G075500.v7.0 | 1.74 | Pectinesterase 2.1 |
| PA | Manes.17G001900.v7.0 | 2.11 | Pectin acetylesterase 7 |
| PG | Manes.13G028600.v7.0 | -2.81 | Polygalacturonase At1g48100 |
| PG | Manes.13G030400.v7.0 | 2.29 | Polygalacturonase-1 non-catalytic subunit beta |
| PG | Manes.05G031300.v7.0 | 1.08 | Probable polygalacturonase |

**Table S6** TFs from all DEGs

| Gene_ID | Family | XX048 Vs NZ199 |
| --- | --- | --- |
| Manes.01G024100.v7.0 | C2C2-CO-like | -3.20 |
| Manes.01G035000.v7.0 | C2C2-Dof | -1.34 |
| Manes.01G054100.v7.0 | bHLH | -1.39 |
| Manes.01G057200.v7.0 | MYB | -1.92 |
| Manes.01G081000.v7.0 | C2H2 | -1.69 |
| Manes.01G083900.v7.0 | MYB-related | -1.11 |
| Manes.01G085800.v7.0 | AP2/ERF-RAV | -3.47 |
| Manes.01G105400.v7.0 | bZIP | -2.46 |
| Manes.01G123100.v7.0 | MYB-related | -1.74 |
| Manes.01G149900.v7.0 | bZIP | -1.43 |
| Manes.01G262100.v7.0 | AP2/ERF-ERF | -8.91 |
| Manes.01G263500.v7.0 | MADS-MIKC | -2.45 |
| Manes.01G270700.v7.0 | SBP | -2.55 |
| Manes.02G011500.v7.0 | WRKY | -3.03 |
| Manes.02G080700.v7.0 | MYB-related | -1.66 |
| Manes.02G147300.v7.0 | zf-HD | -1.02 |
| Manes.02G179200.v7.0 | Trihelix | -1.42 |
| Manes.02G205100.v7.0 | C2H2 | -1.43 |
| Manes.03G010600.v7.0 | C2H2 | -1.46 |
| Manes.03G033200.v7.0 | C2C2-GATA | -1.73 |
| Manes.03G164200.v7.0 | MYB | -3.08 |
| Manes.04G132800.v7.0 | C2C2-GATA | -1.15 |
| Manes.05G041900.v7.0 | MADS-MIKC | -2.41 |
| Manes.05G052900.v7.0 | HB-HD-ZIP | -1.03 |
| Manes.05G157900.v7.0 | TUB | -1.60 |
| Manes.05G158500.v7.0 | zf-HD | -1.05 |
| Manes.05G168600.v7.0 | GARP-G2-like | -1.03 |
| Manes.05G203900.v7.0 | WRKY | -1.04 |
| Manes.06G079700.v7.0 | bZIP | -2.04 |
| Manes.08G031100.v7.0 | OFP | -1.67 |
| Manes.08G099700.v7.0 | C2H2 | -1.36 |
| Manes.09G032800.v7.0 | SBP | -3.12 |
| Manes.09G061200.v7.0 | Trihelix | -1.02 |
| Manes.10G033200.v7.0 | B3 | -2.16 |
| Manes.10G106000.v7.0 | zf-HD | -1.53 |
| Manes.10G129900.v7.0 | MADS-M-type | -1.65 |
| Manes.11G029700.v7.0 | bHLH | -1.53 |
| Manes.11G082100.v7.0 | DBB | -1.13 |
| Manes.11G161000.v7.0 | C2H2 | -1.10 |
| Manes.12G017600.v7.0 | GARP-ARR-B | -3.82 |
| Manes.14G080400.v7.0 | NAC | -2.29 |
| Manes.14G080600.v7.0 | MYB | -1.77 |
| Manes.14G083200.v7.0 | bHLH | -1.46 |
| Manes.14G089600.v7.0 | bHLH | -1.02 |
| Manes.14G122700.v7.0 | BES1 | -1.08 |
| Manes.15G078700.v7.0 | GRAS | -3.05 |
| Manes.15G122300.v7.0 | C2H2 | -1.14 |
| Manes.15G177200.v7.0 | bHLH | -1.35 |
| Manes.17G047500.v7.0 | SBP | -1.32 |
| Manes.18G063000.v7.0 | bHLH | -1.18 |
| Manes.S025300.v7.0 | HB-other | -3.04 |
| Manihot_esculenta_newGene_3087 | NAC | -1.33 |
| Manihot_esculenta_newGene_4274 | MYB | -3.64 |
| Manihot_esculenta_newGene_4782 | bZIP | -1.62 |
| Manihot_esculenta_newGene_4970 | bHLH | -1.97 |
| Manihot_esculenta_newGene_5684 | Trihelix | -1.42 |
| Manes.01G085400.v7.0 | AP2/ERF-ERF | 1.95 |
| Manes.01G133500.v7.0 | OFP | 1.20 |
| Manes.01G228500.v7.0 | WRKY | 1.10 |
| Manes.01G238300.v7.0 | NAC | 2.15 |
| Manes.01G241700.v7.0 | bHLH | 2.09 |
| Manes.02G094900.v7.0 | MYB-related | 1.54 |
| Manes.02G122200.v7.0 | MYB | 1.08 |
| Manes.02G142500.v7.0 | GRAS | 1.14 |
| Manes.02G160100.v7.0 | C2H2 | 1.13 |
| Manes.03G098700.v7.0 | MYB | 1.64 |
| Manes.04G150100.v7.0 | AP2/ERF-ERF | 1.07 |
| Manes.05G026200.v7.0 | AP2/ERF-ERF | 1.06 |
| Manes.05G126800.v7.0 | NAC | 1.58 |
| Manes.05G175700.v7.0 | HB-WOX | 1.08 |
| Manes.06G018700.v7.0 | bHLH | 1.30 |
| Manes.06G041200.v7.0 | HB-KNOX | 1.06 |
| Manes.06G101900.v7.0 | LIM | 1.58 |
| Manes.06G109000.v7.0 | MYB | 2.39 |
| Manes.07G125100.v7.0 | MYB | 1.50 |
| Manes.08G018300.v7.0 | HB-HD-ZIP | 2.76 |
| Manes.08G031200.v7.0 | OFP | 1.42 |
| Manes.09G033400.v7.0 | GARP-G2-like | 2.45 |
| Manes.09G080100.v7.0 | AP2/ERF-AP2 | 1.69 |
| Manes.10G063200.v7.0 | AP2/ERF-AP2 | 2.38 |
| Manes.11G064200.v7.0 | AP2/ERF-ERF | 1.88 |
| Manes.13G077500.v7.0 | NF-YB | 2.17 |
| Manes.13G130800.v7.0 | B3 | 3.47 |
| Manes.13G144400.v7.0 | SBP | 1.11 |
| Manes.14G066200.v7.0 | MYB | 1.89 |
| Manes.14G092600.v7.0 | bZIP | 1.47 |
| Manes.15G032900.v7.0 | HSF | 4.94 |
| Manes.16G079300.v7.0 | HB-BELL | 1.61 |
| Manihot_esculenta_newGene_2726 | HB-HD-ZIP | 2.00 |
| Manihot_esculenta_newGene_3252 | WRKY | 2.02 |
| Manihot_esculenta_newGene_4859 | MYB-related | 2.86 |

**Table S7** Primer sequences of qRT-PCR

| \| Gene_ID \| \| --- \| | Primer name | Primer sequence(5'−3') |
| --- | --- | --- | --- |
| Actin | Actin F | TGATGAGTCTGGTCCATCCA |
|  | Actin R | CCTCCTACGACCCAATCTCA |
| Manes.02G104700 | Manes.02G104700 F | GGCGGCTTCATCGTCTCTAG |
|  | Manes.02G104700 R | CTCGGTCACTGCTCTCCATC |
| Manes.02G142500 | Manes.02G142500 F | TCCTGAATCAACCTCATCATCC |
|  | Manes.02G142500 R | AAGACTCCAATAGCCTCTCCAT |
| Manes.04G094200 | Manes.04G094200 F | GTGCTCGTGTTCTTGTTGTGT |
|  | Manes.04G094200 R | TGGACGCTCAATGGAAGTATCA |
| Manes.04G096600 | Manes.04G096600 F | GCAGACGATTGGGCTACGA |
|  | Manes.04G096600 R | CTCACAGAACTTGGCATTCACT |
| Manes.06G034800 | Manes.06G034800 F | TGGTGGACTATCTCTCGGACTT |
|  | Manes.06G034800 R | ATGAACGGTGGCATTGTAATGG |
| Manes.07G076800 | Manes.07G076800 F | GGCTGTGGAGTGGATGCTTA |
|  | Manes.07G076800 R | GGTCAAGGAGAAGAGGCACTT |
| Manes.08G066200 | Manes.08G066200 F | ATGTTCGCACTGGACACTGT |
|  | Manes.08G066200 R | GCTGCCACCATCGGATTCT |
| Manes.08G128900 | Manes.08G128900 F | GCTCTTCCTTCCACCGAGTT |
|  | Manes.08G128900 R | GCAGCAGCCGCAATGAATT |
| Manes.11G088400 | Manes.11G088400 F | TTCGTTTGCCTGCCGTGAT |
|  | Manes.11G088400 R | CCTGGAAGCAAGGATGGTTCA |
| Manes.17G047500 | Manes.17G047500 F | TGAGGAGGATGAGGAGGATGAA |
|  | Manes.17G047500 R | TGGAACTCGCACACCTTATGG |
| newGene_338 | newGene_338 F | CTTGCTGTTCAGGTCACTAAGT |
|  | newGene_338 R | CGGTGGCATTGTAATGGAGAG |
